# Association of hemorrhoidal disease with dementia risk: a nationwide cohort study

**DOI:** 10.3389/fneur.2025.1655944

**Published:** 2025-10-02

**Authors:** Ho Geol Woo, Moo-Seok Park, Ju-Young Park, Hyejin Chun, Tae-Jin Song

**Affiliations:** ^1^Department of Neurology, Kyung Hee University College of Medicine, Seoul, Republic of Korea; ^2^Department of Neurology, Seoul Hospital, Ewha Womans University College of Medicine, Seoul, Republic of Korea; ^3^Department of Statistics, Yeungnam University, Gyeongsan, Gyeongbuk, Republic of Korea; ^4^Department of Family Medicine, Seoul Hospital, Ewha Womans University College of Medicine, Seoul, Republic of Korea; ^5^Graduate Program in System Health Science and Engineering, Ewha Womans University, Seoul, Republic of Korea

**Keywords:** hemorrhoids, dementia, epidemiology, veins, treatment

## Abstract

**Introduction:**

Research on the association between hemorrhoidal diseases (HDs) and dementia is limited. We explored this relationship in a population-based longitudinal cohort and proposed that individuals with HD may experience a higher incidence of dementia.

**Methods:**

Our study included 381,031 participants drawn from results from the South Korean health-screening cohort database, between 2005 and 2010. HD was identified based on at least two claims using the International Classification of Diseases, Tenth Revision (ICD-10) code I84. We used propensity score matching (PSM) to categorize the participants into two groups based on the presence or treatment of HD. The primary outcome was the incidence of all-cause dementia as determined by two or more claims with ICD-10 codes (F00-03, G30, and G31). Secondary outcomes included the occurrence of Alzheimer’s (F00 or G30) and vascular dementia (F01).

**Results:**

Over a median follow-up period of 15.49 years (interquartile range: 12.21–18.77 years), the cumulative incidence of all-cause dementia was 80,488 cases (22.47%). Multivariate analysis showed that the group with HD consistently had a higher incidence of all-cause dementia than the group without HD after PSM (hazard ratio [HR], 1.243; 95% confidence interval [CI], 1.199–1.288). Participants who underwent surgical procedures or treatment for HD revealed a significantly lower incidence of all-cause dementia after PSM (HR, 0.925; 95% CI, 0.872–0.981).

**Discussion:**

This study revealed a significantly higher incidence of all-cause dementia among participants with hemorrhoidal disease, suggesting that while hemorrhoidal disease may not directly cause dementia, it may serve as a marker of an underlying systemic condition that increases dementia risk.

## 1 Introduction

Hemorrhoids are naturally occurring vascular structures located in the lower rectum and anal canal. They are essential for maintaining continence (1). However, the term “hemorrhoids” commonly refers to a pathological condition involving the abnormal displacement of this tissue, known as hemorrhoidal disease (1). This condition affects nearly 40% of adults and can cause considerable discomfort, disrupt daily functioning, and negatively impact the quality of life (2, 3). Hemorrhoidal disease poses a significant clinical challenge and has notable socioeconomic consequences, exerting pressure on healthcare resources (4).

Dementia refers to a group of disorders characterized by persistent and worsening memory decline, cognitive dysfunction, and behavioral alterations that interfere with everyday functioning (5). Alzheimer’s disease (AD) and vascular dementia (VD) account for most dementia cases. Although VD is traditionally viewed as a consequence of stroke and vascular disease, increasing evidence indicates that vascular risk factors play a role in AD (5). The exact cause of dementia remains unclear; however, genetic predisposition, environmental factors, neuroinflammation, and vascular damage appear to contribute to its development (6). Further research is necessary to explore the associations and risk factors associated with dementia.

Although hemorrhoidal disease is relatively common, it may also be a clinical sign of compromised vascular integrity. In addition to other chronic venous conditions, hemorrhoidal diseases may be associated with an increased risk of cardiovascular diseases (7). Furthermore, damage to the cerebral venous integrity is a well-known pathological mechanism of dementia (8). Therefore, hemorrhoidal disease and dementia may share several risk factors, and are possibly related (8, 9). However, there has been limited research on the association between the presence of hemorrhoidal disease and the risk of dementia. We proposed that hemorrhoidal disease is associated with a heightened risk of developing dementia. This study aimed to explore the relationship between the occurrence and management of hemorrhoidal disease and the likelihood of dementia, using a nationwide longitudinal dataset from the general population.

## 2 Materials and methods

### 2.1 Data source

The Korean National Health Insurance System (NHIS) provides an extensive database containing information on demographics, socioeconomic status, medical diagnoses, and treatment methods. It also includes data from national health screening and healthcare facilities (10). According to the NHIS recommendations, individuals enrolled in the system are encouraged to undergo regular biennial health screening. This study made use of data from the NHIS-National Health Screening Cohort (NHIS-HEALS) (11, 12). The NHIS-HEALS cohort comprises of data from 10% of individuals, randomly selected, who underwent health examinations between 2002 and 2019. This cohort includes participants over the age of 40 who participated in the NHIS health-screening programs. From this cohort, we gathered data including demographic information, height, weight, household income, smoking and alcohol habits, physical activity, existing medical history, and health conditions. This study adhered to the guidelines of the Declaration of Helsinki and was approved by the Institutional Review Board of Ewha Womans University Seoul Hospital (EUMC 2024-03-006). The requirement for informed consent was waived owing to the retrospective nature of the study and the minimal risk to the patients.

### 2.2 Study population

**Figure 1:**
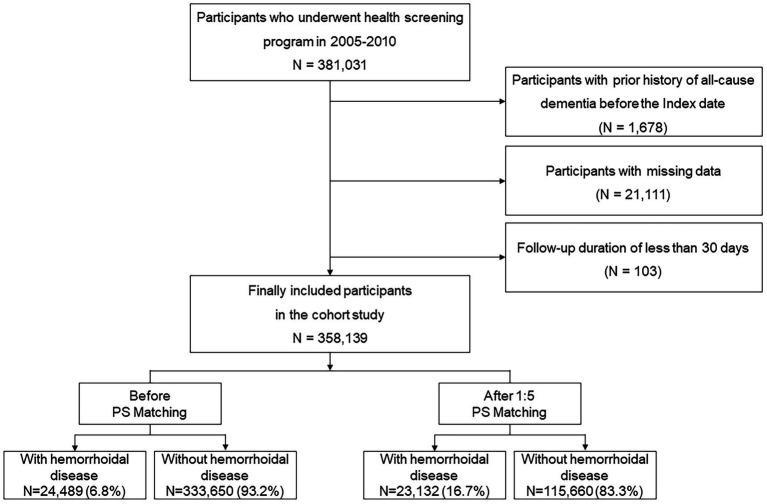
Flow chart of inclusion and exclusion criteria.

Using data from the NHIS-HEALS database, we selected 381,031 participants between 2005 and 2010, who were then followed up through to December 31, 2019. Those with a prior history of dementia, that is 1,678 individuals with International Classification of Diseases-10 (ICD-10) codes F00, F01, F02, F03, G30, or G31 were excluded based on records from January 1, 2002, to December 31, 2010. Additionally, 21,111 participants with missing or incomplete data, and 103 individuals with less than 30 days of follow-up, were excluded to minimize reverse causation. The final study population comprised of 358,139 individuals (Figure 1).

### 2.3 Definition of hemorrhoidal disease

The presence of hemorrhoidal disease was identified when participants had at least two separate medical claims with ICD-10 code I84, following the criteria of a previous study (13). Surgical and procedural treatments for hemorrhoids were identified using the following procedure codes: Q3012 for thrombosed hemorrhoid surgery, Q3013 for hemorrhoidectomy, Q3014 for surgery on strangulated circumferential hemorrhoids, Q3015 for thrombectomy or excision of hemorrhoidal skin tags, Q3016 for treatments including coagulation, cauterization, sclerotherapy, or rubber band ligation, and Q3017 for circular stapled hemorrhoidectomy (13).

### 2.4 Definition of outcomes

The main outcome of this study was the occurrence of all-cause dementia, including AD, VD, and other forms of dementia, as identified by the ICD-10 diagnostic codes F00-F03, G30, and G31. An event was considered an outcome when it corresponded to these ICD-10 codes, and there were records of prescribed anti-dementia medications such as donepezil, galantamine, rivastigmine, and memantine. This algorithm for defining dementia cases is acceptable because of its high level of accuracy, as evidenced by a 94.7% positive predictive value (14). Secondary outcomes were AD (ICD-10 codes F00 or G30) and VD (ICD-10 code F01). Follow-up was performed until either death, the first incidence of all-cause dementia or December 31, 2019.

### 2.5 Definition of covariates

Age, sex, body mass index (BMI), household income, smoking status, alcohol consumption, regular physical activity, hypertension, diabetes mellitus, dyslipidemia, stroke, myocardial infarction, chronic obstructive pulmonary disease (COPD), renal disease, liver disease, cancer, social determinants of health (SDoH), inflammatory bowel disease (IBD), and Charlson Comorbidity Index (CCI) were investigated as covariates. SDoH was defined using low-income status and claims of ICD-10 codes, including depression, anxiety-, and stress-related disorders. Detailed definitions of these covariates are provided in Supplementary Methods 1 (15–18).

### 2.6 Statistical analysis

A comparison of the baseline characteristics between the participants in the hemorrhoidal disease group and those in the non-hemorrhoidal disease group was performed using an independent t-test for continuous variables and a chi-squared test (or Fisher’s exact test) for categorical variables. To ensure balanced baseline characteristics and minimize potential confounding between the two groups, a 1:5 propensity score matching (PSM) approach was employed (13, 19). Propensity scores were estimated using a multivariate logistic regression model including demographic, lifestyle, and clinical variables (age, sex, BMI, household income, smoking status, alcohol consumption, regular physical activity, hypertension, diabetes mellitus, dyslipidemia, stroke, myocardial infarction, COPD, renal disease, liver disease, cancer, and CCI) Supplementary Methods 2. The effectiveness of matching was evaluated using the standardized mean difference (SMD), with an absolute SMD value below 0.1 indicating an acceptable level of balance (20, 21). The calculation of the SMD for a categorical variable is described in Supplementary Methods 3.

To analyze the incidence risk of all-cause dementia, AD, and VD, we used Kaplan–Meier survival curves and tested the differences between the hemorrhoidal and non-hemorrhoidal disease groups using log-rank tests. Cox proportional hazards models were used to calculate the hazard ratios (HRs) and 95% confidence intervals (CIs). We used the Grambsch and Therneau test based on Schoenfeld residuals and confirmed that the proportional hazards assumption was reasonably satisfied in our model (22). Additionally, we analyzed the Wald Chi-square test results from the Cox proportional hazards model, including hemorrhoid treatment status as a covariate, based on the cohort before and after PSM. These were visualized using forest plots.

Further subgroup analyses were performed to assess the association between the presence of hemorrhoidal disease and all-cause dementia according to demographic data and covariates, with *p*-values for interaction. Additionally, we performed Wald chi-square statistics on the Cox model before and after PSM. To assess the relationship between the severity and treatment of hemorrhoidal disease and the risk of developing dementia, a sensitivity analysis was conducted to examine whether surgical intervention or other treatments for hemorrhoidal disease were associated with the incidence of all-cause dementia, AD, and VD. This analysis was conducted using Cox regression, with pre- and post- 1:1 PSM. Furthermore, we conducted a mediation analysis based on the Cox proportional hazards model to evaluate whether the effect of hemorrhoidal disease on dementia is mediated through a selected dementia risk factor considering the SDoH variable (Supplementary Methods 4 and Supplementary Figure 1) (23). Statistical analyses were conducted using SAS software (version 9.4; SAS Institute Inc., Cary, NC, USA) and R version 4.2.1 (R Foundation for Statistical Computing, Vienna, Austria). A two-sided *p*-value of less than 0.05 was considered statistically significant for all tests.

## 3 Results

### 3.1 Baseline characteristics of participants

Table 1 shows the baseline characteristics based on the presence of hemorrhoidal disease. The average age of the participants was 54.4 ± 9.7 years, with males comprising 53.7% of the population. Patients diagnosed with hemorrhoidal disease tended to be younger and of male gender. In addition, they were less likely to have a history of smoking, heavy alcohol consumption, hypertension, or diabetes mellitus. Conversely, individuals with hemorrhoidal disease more commonly exhibited comorbid conditions such as dyslipidemia, stroke, myocardial infarction, COPD, kidney disease, liver disease, cancer, SDoH, and IBD, and had higher CCI scores (Table 1). Following PSM, the groups with and without hemorrhoidal disease were well matched across the covariates (Table 1).

**Table 1:** Baseline characteristics of study participants.

Variable	Before PSM, *N* = 358,139				After 1:5 PSM, *N* = 138,792		
	Total	Hemorrhoidal disease (−)	Hemorrhoidal disease (+)	*p*-value	Hemorrhoidal disease (−)	Hemorrhoidal disease (+)	SMD^*^
		Mean ± SD, *N* (%)	Mean ± SD, *N* (%)		Mean ± SD, *N* (%)	Mean ± SD, *N* (%)	
Number	358,139	333,650	24,489		115,660	23,132	
Age, years	54.4 ± 9.7	54.4 ± 9.7	53.8 ± 8.8	<0.001	53.7 ± 9.5	53.8 ± 8.9	−0.013
Sex				<0.001			−0.001
Female	165,815 (46.3)	155,832 (46.7)	9,983 (40.8)		48,675 (42.1)	9,726 (42.1)	
Male	192,324 (53.7)	177,818 (53.3)	14,506 (59.2)		66,985 (57.9)	13,406 (57.9)	
Body mass index (kg/m^2^)	24.0 ± 3.0	24.0 ± 3.0	24.0 ± 2.8	0.620	23.9 ± 2.9	23.9 ± 2.8	−0.002
Household income				<0.001			0.001
Low	108,030 (30.2)	101,970 (30.6)	6,060 (24.8)		29,333 (25.4)	5,859 (25.3)	
Middle	130,145 (36.3)	121,297 (36.4)	8,848 (36.1)		41,791 (36.1)	8,374 (36.2)	
High	119,964 (33.5)	110,383 (33.0)	9,581 (39.1)		44,536 (38.5)	8,899 (38.5)	
Smoking status				<0.001			0.001
Never	244,775 (68.4)	227,579 (68.2)	17,196 (70.2)		81,459 (70.4)	16,278 (70.4)	
Former	30,609 (8.6)	27,939 (8.4)	2,670 (10.9)		12,179 (10.5)	2,426 (10.5)	
Current	82,755 (23.0)	78,132 (23.4)	4,623 (18.9)		22,022 (19.1)	4,428 (19.1)	
Alcohol consumption (days/week)				<0.001			−0.001
None	208,935 (58.3)	194,922 (58.4)	14,013 (57.2)		66,739 (57.7)	13,361 (57.8)	
1–2 times	109,414 (30.6)	101,275 (30.4)	8,139 (33.2)		37,964 (32.8)	7,569 (32.7)	
3–4 times	24,188 (6.8)	22,648 (6.8)	1,540 (6.3)		7,231 (6.4)	1,437 (6.2)	
≥5 times	15,602 (4.3)	14,805 (4.4)	797 (3.3)		3,726 (3.1)	765 (3.3)	
Regular physical activity (days/week)				<0.001			0.001
None	198,854 (55.5)	186,968 (56.0)	11,886 (48.5)		57,249 (49.5)	11,434 (49.4)	
1–4 days	123,151 (34.4)	113,154 (33.9)	9,997 (40.8)		46,180 (39.9)	9,223 (39.9)	
≥5 days	36,134 (10.1)	33,528 (10.1)	2,606 (10.7)		12,231 (10.6)	2,475 (10.7)	
Comorbidities							
Hypertension	88,684 (24.8)	83,396 (25.0)	5,288 (21.6)	<0.001	25,058 (21.7)	5,032 (21.8)	−0.003
Diabetes mellitus	37,488 (10.5)	35,636 (10.7)	1,852 (7.6)	<0.001	8,944 (7.7)	1,814 (7.8)	−0.004
Dyslipidemia	50,625 (14.1)	44,907 (13.5)	5,718 (23.4)	<0.001	24,169 (20.9)	4,887 (21.1)	−0.006
Stroke	766 (0.2)	686 (0.2)	80 (0.3)	<0.001	313 (0.3)	61 (0.3)	0.002
Myocardial infarction	389 (0.1)	358 (0.1)	31 (0.1)	0.376	149 (0.1)	29 (0.1)	0.000
COPD	62,902 (17.6)	56,455 (16.9)	6,447 (26.3)	<0.001	28,622 (24.8)	5,703 (24.7)	0.002
Renal disease	7,464 (2.1)	6,759 (2.0)	705 (2.9)	<0.001	3,027 (2.6)	629 (2.7)	−0.006
Liver disease	43,674 (12.2)	38,299 (11.5)	5,375 (22.0)	<0.001	22,247 (19.2)	4,506 (19.5)	−0.006
Cancer	10,943 (3.1)	9,676 (2.9)	1,267 (5.2)	<0.001	4,999 (4.3)	1,042 (4.5)	−0.009
SDoH	4,762 (1.3)	4,215 (1.3)	547 (2.2)	<0.001	1,453 (1.3)	522 (2.3)	−0.088
IBD	2,529 (0.7)	2,137 (0.6)	392 (1.6)	<0.001	889 (0.8)	372 (1.6)	−0.090
Charlson comorbidity index				<0.001			0.004
0	313,901 (93.1)	291,415 (93.1)	22,486 (93.2)		107,875 (93.3)	21,557 (93.2)	
1	20,783 (6.2)	19,276 (6.2)	1,507 (6.2)		7,120 (6.2)	1,443 (6.2)	
≥2	2,223 (0.7)	2,089 (0.7)	134 (0.6)		665 (0.5)	132 (0.6)	

COPD, chronic obstructive pulmonary disease; IBD, inflammatory bowel disease; *N*, number; PSM, propensity score matching; SD, standard deviation; SDoH, social determinants of health; SMD, standardized mean difference.

* All standardized mean difference values were <0.1 in the propensity score matched cohort.

### 3.2 Association of presence of hemorrhoidal disease with dementia

**Figure 2:**
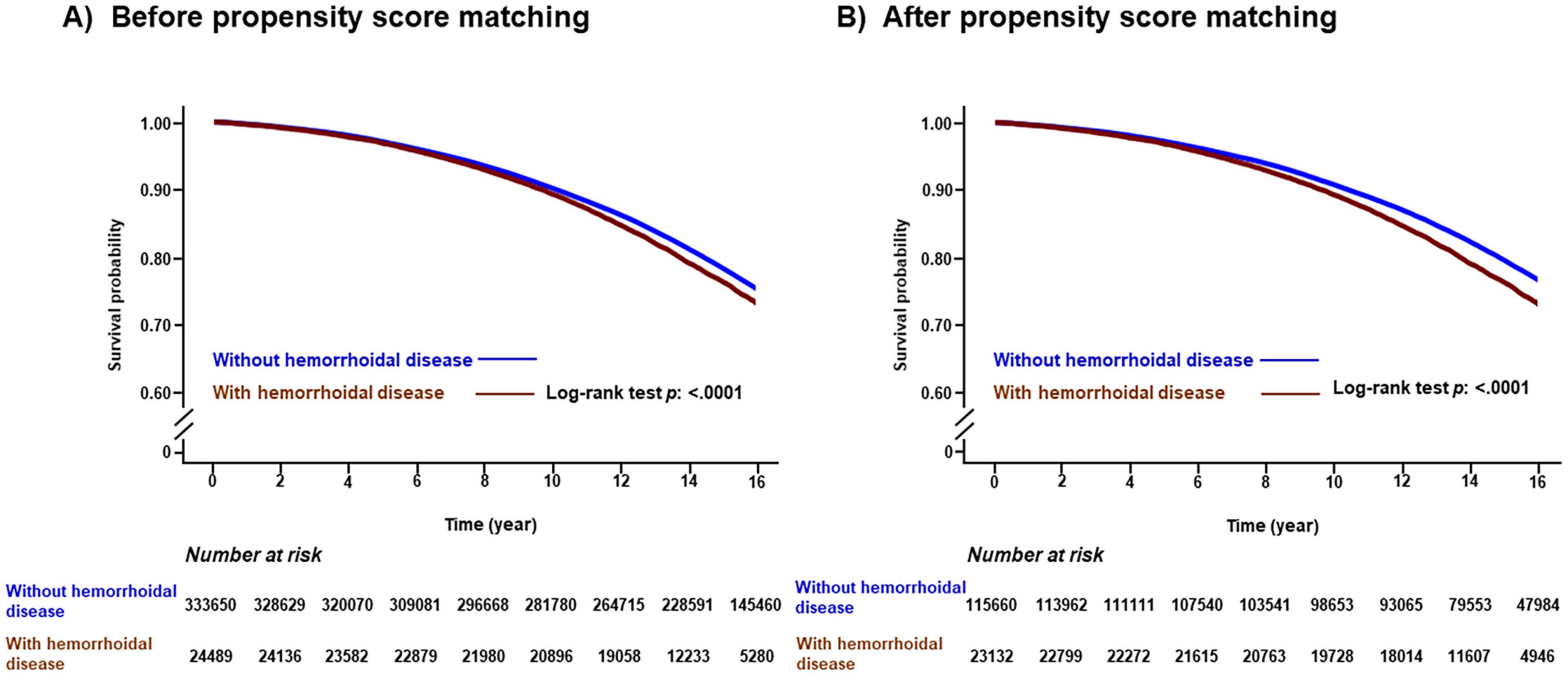
Kaplan-Meier survival curves of all-cause dementia according to presence of hemorrhoidal disease before propensity score matching **(A)** and after propensity score matching **(B)**.

**Figure 3:**
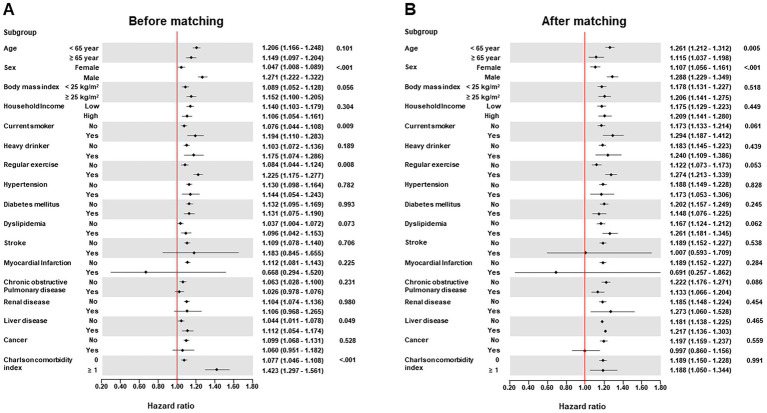
Forest plots of subgroup analysis according to demographic data and comorbidities for the association of hemorrhoidal disease with incidence risk of all-cause dementia before propensity score matching **(A)** and after propensity score matching **(B)**.

During a median follow-up period of 15.49 years (interquartile range: 12.21–18.77 years), the cumulative incidence of all-cause dementia was 80,488 (22.47%), including 38,094 cases of AD (10.64%) and 15,198 cases of VD (4.24%). The Kaplan–Meier survival curves for the occurrence of dementia according to the presence of hemorrhoidal disease are shown in Figure 2. Participants had an increased risk of all-cause dementia according to the presence of hemorrhoidal disease (log-rank test, *p* < 0.001) regardless of PSM. In the multivariate Cox regression analysis, the hemorrhoidal disease group consistently showed an increased risk of all-cause dementia compared with the group without hemorrhoidal disease before PSM (HR: 1.228; 95% CI: 1.194–1.263; *p* < 0.001) and after PSM (HR: 1.243; 95% CI: 1.199–1.288; *p* < 0.001; Table 2 and Supplementary Table 1). In the Wald Chi-square test, the global tests for the full model (including 26 covariates) were highly significant for all outcomes before PSM (Wald *χ*^2^ = 79,669.454 for all-cause dementia, *p* < 0.001) and after PSM (Wald *χ*^2^ = 32,255.940 for all-cause dementia, *p* < 0.001; Supplementary Tables 2, 3). Additionally, these positive associations were consistently noted in AD before PSM (HR: 1.073; 95% CI: 1.028–1.121; *p* < 0.001) and after PSM (HR: 1.075; 95% CI: 1.012–1.142; *p* < 0.001), and VD before PSM (HR: 1.130; 95% CI: 1.103–1.157; *p* < 0.001) and after PSM (HR: 1.086; 95% CI: 1.020–1.152; *p* < 0.001; Table 2, Supplementary Tables 4, 5). Furthermore, strong global significance was noted in AD before PSM (Wald *χ*^2^ = 57,133.027, df = 26, *p* < 0.001) and after PSM (Wald *χ*^2^ = 22,102.189, df = 26, *p* < 0.001), and VD before PSM (Wald *χ*^2^ = 16,841.816, df = 26, *p* < 0.001) and after PSM (Wald *χ*^2^ = 6,655.687, df = 26, *p* < 0.001; Supplementary Tables 2, 3). In the subgroup analysis, the link between hemorrhoidal disease and an elevated risk of all-cause dementia remained consistent across all covariate categories (Figure 3, Supplementary Tables 6, 7).

**Table 2:** Results of Cox regression analysis for the association of hemorrhoidal disease with incidence risk of dementia.

Variable	Before PSM *N* = 358,139			After 1:5 PSM *N* = 138,792		
	Incidence rate (per 100,000 person-years)	Crude HR (95%CI)	Adjusted HR (95%CI)	Incidence rate (per 100,000 person-years)	Crude HR (95%CI)	Adjusted HR (95%CI)
All-cause dementia	1,633.404	1.111 (1.081–1.142)	1.228 (1.194–1.263)	1,557.404	1.188 (1.152–1.226)	1.243 (1.199–1.288)
Alzheimer’s disease	747.482	1.115 (1.076–1.155)	1.073 (1.028–1.121)	671.973	1.038 (0.990–1.088)	1.075 (1.012–1.142)
Vascular dementia	292.398	1.053 (1.023–1.084)	1.130 (1.103–1.157)	268.584	1.045 (0.972–1.124)	1.086 (1.020–1.152)

CI, confidence interval; HR, hazard ratio; *N*, number; PSM, propensity score matching. Values from the multivariable Cox regression models were adjusted for age, sex, body mass index, household income, smoking status, alcohol consumption, regular physical activity, comorbidities, social determinants of health, inflammatory bowel disease and Charlson comorbidity index.

### 3.3 Association of treatment/procedure of hemorrhoidal disease with dementia

Supplementary Table 8 outlines the clinical characteristics before and after PSM based on whether the participants underwent surgical procedures or treatments for hemorrhoidal disease. The specific frequencies of these procedures and treatments are shown in Supplementary Table 9. Notably, participants who underwent surgical intervention or treatment for hemorrhoidal disease exhibited a significantly reduced risk of developing all-cause dementia (HR: 0.925; 95% CI: 0.872–0.981; *p* = 0.012), AD (HR: 0.901; 95% CI: 0.823–0.987; *p* = 0.007), and VD (HR: 0.848; 95% CI: 0.736–0.976; *p* = 0.003), even after PSM in multivariate Cox regression analysis (Table 3, Supplementary Tables 10–12). In addition, the Wald Chi-square test results showed that hemorrhoid treatment status was a significant predictor of dementia, both before and after PSM (Supplementary Tables 13, 14). Moreover, in the mediation analysis, our findings were significant, even when considering SDoH as a mediator (Supplementary Tables 15, 16). There was no significant multicollinearity in the association between hemorrhoidal disease and dementia among the covariates (Supplementary Table 17).

**Table 3:** Results of Cox regression analysis for the association of surgical procedure/treatment for hemorrhoidal disease with incidence risk of dementia.

Variable	Before PSM *N* = 24,489			After 1:1 PSM *N* = 18,330		
	Incidence rate (per 100,000 person-years)	Crude HR (95%CI)	Adjusted HR (95%CI)	Incidence rate (per 100,000 person-years)	Crude HR (95%CI)	Adjusted HR (95%CI)
All-cause dementia	1,692.232	0.660 (0.626–0.696)	0.904 (0.856–0.954)	1,876.238	0.939 (0.886–0.996)	0.925 (0.872–0.981)
Alzheimer’s disease	671.663	0.570 (0.525–0.620)	0.911 (0.837–0.992)	757.833	0.905 (0.826–0.990)	0.901 (0.823–0.987)
Vascular dementia	275.762	0.578 (0.508–0.657)	0.865 (0.758–0.988)	306.753	0.846 (0.734–0.974)	0.848 (0.736–0.976)

CI, confidence interval; HR, hazard ratio; *N*, number; PSM, propensity score matching. Values from the multivariable Cox regression models were adjusted for age, sex, body mass index, household income, smoking status, alcohol consumption, regular physical activity, comorbidities, social determinants of health, inflammatory bowel disease and Charlson comorbidity index.

## 4 Discussion

Our study revealed that in a longitudinal setting, the presence of hemorrhoidal disease was associated with the incidence of all-cause dementia, AD, and VD, regardless of PSM. Moreover, our study has several strengths that distinguish it from previous studies. To reduce bias, we used a 1:5 PSM approach for the general population in a longitudinal setting. Our findings confirmed that the presence of hemorrhoidal disease was significantly associated with an increased risk of all-cause dementia, including AD and VD. Furthermore, surgical procedures or treatments for hemorrhoidal diseases may be associated with a lower risk of all-cause dementia, AD, and VD.

There is increasing focus on the role of hemorrhoidal diseases and their association with neuropsychiatric disorders, cardiovascular diseases, and risk factors. Previous studies have suggested that older age, current smoking status, and the presence of hypertension, are associated with hemorrhoidal disease (9). Moreover, increased incidences of peripheral artery occlusive disease and coronary artery disease have been observed in patients with hemorrhoidal disease compared to those without hemorrhoidal disease (7, 24). Furthermore, obesity, abdominal obesity, and depression may be risk factors for hemorrhoidal disease (25). Hemorrhoidal disease is genetically associated with psychiatric symptoms such as depression, bipolar disorder, anxiety disorders, and schizophrenia. However, no studies have examined hemorrhoidal disease and cognitive decline, including AD and VD (26). In our study, we found that the presence of hemorrhoidal disease was associated with an increased risk of dementia, with consistent results across subtypes, including AD and VD. These results provide valuable insights that contribute to the growing body of evidence on the relationship between hemorrhoidal diseases and other health conditions.

A direct relationship between hemorrhoidal diseases and dementia has not yet been clearly established. However, several potential pathophysiologies may be related to these two diseases. First, hemorrhoidal diseases and dementia are associated with several risk factors. According to previous studies, obesity and history of pregnancy are associated with hemorrhoidal disease (25, 27). Additionally, diabetes treatment with metformin reduces the risk of hemorrhoids (28). All-cause dementia is associated with obesity (29, 30), diabetes mellitus (31), and pregnancy (32). The overlap of these risk factors supports the conclusion that an association exists between these two diseases.

Second, the development of dementia may occur through a pro-inflammatory and dysregulated gut–brain axis process. Hemorrhoidal diseases typically cause localized inflammation, but in severe cases it may serve as a source of chronic inflammatory mediators that can enter the systemic circulation through the gut–brain axis, thereby inducing systemic inflammation (33–35). In the past decade, a persistent inflammatory response in the brain has been recognized as a pathological feature of dementia (36). Previous research in both humans and animals indicates that systemic inflammation occurring outside the central nervous system, may contribute significantly to neurodegeneration, the pathology of AD, and cognitive decline in older adults (37). Therefore, severe hemorrhoidal disease can trigger a systemic inflammatory response, including the inflammation of the central nervous system, which may increase the risk of dementia (38). Moreover, hemorrhoidal diseases are associated with gut microbiota dysbiosis, which may contribute to increased intestinal permeability and systemic inflammatory responses (39–41). Such systemic inflammation is known to promote the development of dementia (42). A recent study suggested a causal relationship between gut microflora and the occurrence of dementia and its subtypes, including AD and VD (43). Thus, rather than being a direct cause of dementia, hemorrhoidal diseases may be a sign of a systemic state that causes dementia.

In our study, population who underwent surgical intervention for hemorrhoidal disease—potentially representing a cohort with more severe disease—exhibited a lower risk of developing dementia compared to those who did not receive treatment. While this finding may suggest that proactive management of hemorrhoidal disease could mitigate dementia risk, it is more plausibly attributable to the health-conscious behaviors of individuals who seek surgical care. These individuals may have better access to healthcare resources and engage in healthier lifestyles, factors that are not fully captured by the measured covariates. Thus, the observed association may reflect residual confounding by behavioral characteristics rather than a direct effect of the surgical intervention. That is, because the possibility of healthy user bias cannot be entirely excluded in our analysis, warranting cautious interpretation of the findings.

Our study has several limitations. First, our findings may be affected by ethnic bias, which could limit their applicability to other demographic groups. Therefore, further studies involving diverse racial and ethnic populations are essential to enhance generalizability. Second, despite conducting a sub-analysis of participants with hemorrhoidal disease who underwent surgical procedures or treatment, reliance on claims data limited our ability to verify imaging findings or precisely categorize the severity and type of hemorrhoidal disease. Therefore, we were unable to assess whether dementia risk varies according to the severity of hemorrhoidal disease in a dose–response manner. Additionally, although our study provides evidence that hemorrhoidal disease is associated with dementia, given that the effect size is relatively small, caution is warranted in interpreting this finding. Third, because the sample size was reasonably large, small or even trivial effects may be statistically significant. Although the associations between hemorrhoidal disease and AD or VD, were statistically significant, the magnitudes of these associations, such as hazard ratios, were not large. In particular, the effectiveness of treatments for hemorrhoidal diseases in reducing the risk of dementia is limited. Therefore, the results of this study should be interpreted with caution. Finally, although this was a nationwide cohort study, its retrospective nature presented challenges in establishing clear cause-and-effect relationships.

In conclusion, this study revealed a significantly higher incidence of all-cause dementia among participants with hemorrhoidal disease. This finding suggests that hemorrhoidal disease may be a sign of a systemic state that causes dementia.

## Data Availability

The datasets presented in this article are not readily available because the data used in this study are available from the National Health Insurance Service-National Health Screening Cohort (NHIS-HEALS) database. However, restrictions apply to the public availability of the data used under license for the current study. Requests for access to NHIS data can be made through the National Health Insurance Sharing Service homepage (http://nhiss.nhis.or.kr/bd/ab/bdaba021eng.do). To access the database, a completed application form, research proposal, and application for approval from the Institutional Review Board should be submitted to the Inquiry Committee of Research Support at the NHIS for review. Requests to access the datasets should be directed to http://nhiss.nhis.or.kr/bd/ab/bdaba021eng.do. Our dataset number is NHIS-2024-10-2-145.
