# Supplementary figures and images for: Association of hemorrhoidal disease with dementia risk: a nationwide cohort study
Source: Front Neurol. 2025 Oct 2;16:1655944. doi: 10.3389/fneur.2025.1655944 (PMC12527892; doi:10.3389/fneur.2025.1655944)

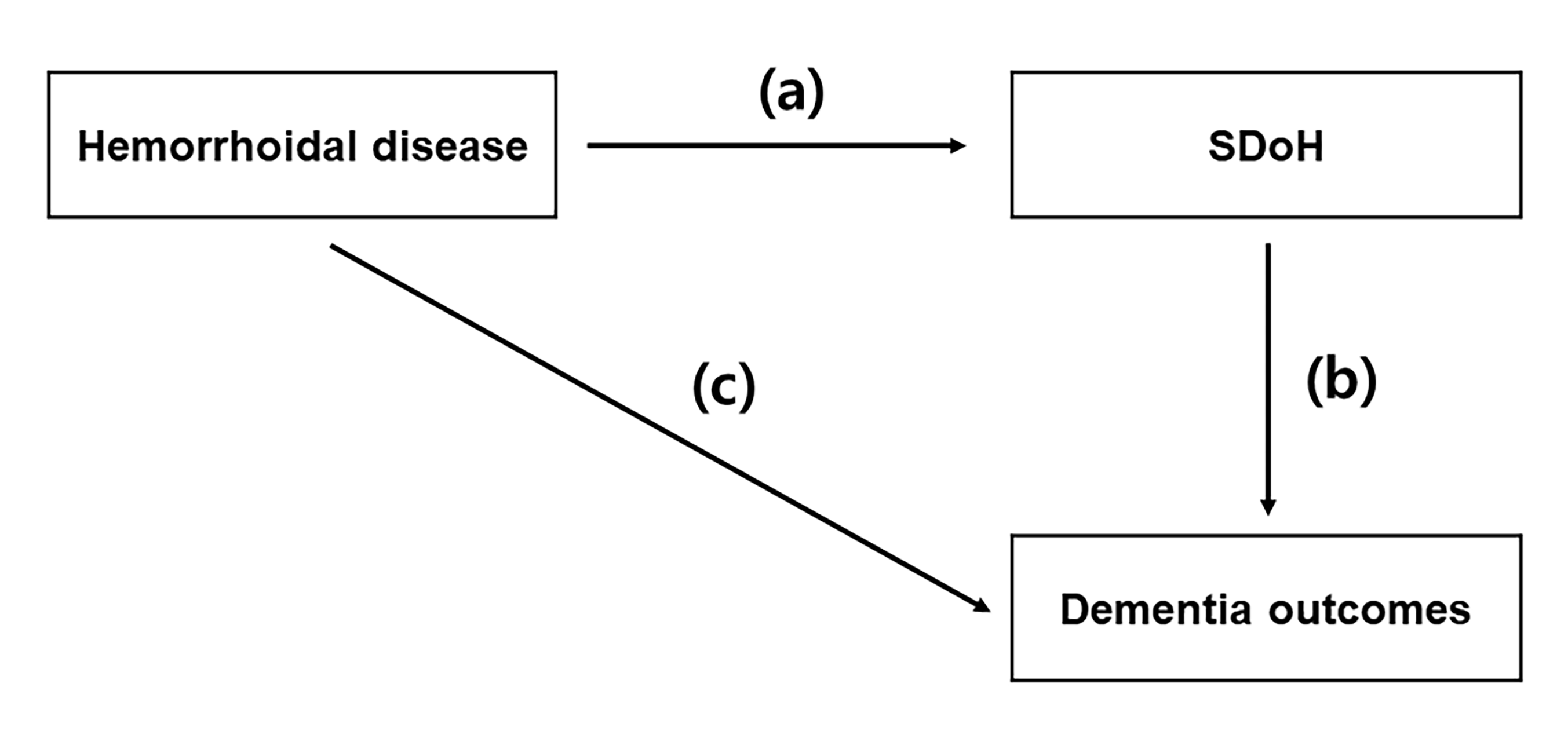

Supplement: Supplementary file 1 [file Image_1.TIF]
