# Supplementary material for: Association of hemorrhoidal disease with dementia risk: a nationwide cohort study
Source: Front Neurol. 2025 Oct 2;16:1655944. doi: 10.3389/fneur.2025.1655944 (PMC12527892; doi:10.3389/fneur.2025.1655944)
Supplement: Supplementary file 2 [file Presentation_1.PDF]

Supplementary Method 1

Definition of covariates

Smoking history (never, former, and current)

Definition and measurement of variables like usual smoking habits were obtained by questionnaire in the health examination program. Smoking status was used to categorize participants into three groups: none, former smoker, and current smoker. Current smoker was classified according to the WHO definition as a person who has smoked more than five packs (100 cigarettes) in a lifetime and smoked daily or occasionally for the last 28 days. Former smoker was defined as a person who had smoked more than 100 cigarettes in a lifetime and had not smoked in the last 28 days (1).

Alcohol consumption (none, moderate, and heavy)

Definition and measurement of variables like usual alcohol consumption were obtained by questionnaire in the health examination program. Alcohol consumption was categorized into three groups: none, moderate drinker, and heavy drinker. Differentiation between moderate and heavy drinker was based on whether a patient usually takes more than 14 drinks/7 drinks per week for men/women. The drinks were calculated by multiplying the average drinking frequency per week by the number of drinks per occasion (2).

Physical activity (low, moderate)

Physical activity was assessed using the Korean version of the International Physical Activity Questionnaire-short form. We created composite physical activity based on Metabolic Equivalent Task (MET)-minutes/week (walking: 3.3 METs; moderate physical activity: 4.0 METs; vigorous physical activity: 8.0 METs), which was categorized as follows based on

total physical activity metabolic equivalents: low (< 600 METs), moderate (600–2,999 METs), and vigorous (≥ 3,000 METs) (3).

Comorbidities

Hypertension

Hypertension was defined as using at least one claim of ICD-10 code(I10-15) with the prescription of an anti-hypertensive agent, claims of ICD-10 code(I10-15) more than two times, a systolic blood pressure of ≥140 mmHg and a diastolic blood pressure of ≥90 mmHg or positive checking in self-report questionnaire on hypertension in the health examination program (4).

Diabetes mellitus

Diabetes mellitus was as defined using at least one claim of ICD-10 code(E11-14) with the prescription of an anti-diabetic agent, claims of ICD-10 code(E11-14) more than two times, fasting serum glucose concentration of ≥7.0 mmol/L or positive checking in self-report questionnaire on diabetes mellitus in the health examination program (4).

Dyslipidemia

Dyslipidemia was defined as using at least one claim of ICD-10 code(E78) with the prescription of an anti-dyslipidemic agent, claims of ICD-10 code(E78) more than two times or total cholesterol level of ≥240 mg/dL (4).

Stroke

Stroke was defined as using claims of ICD-10 codes (I60 – I63) with primary diagnosis, admission≥1 times (≥3 days) and brain imaging (CT or MRI) ≥1 times (4).

Myocardial infarction

Myocardial infarction was defined as using claims of ICD–10 codes (I21, I22) with primary diagnosis with admission of one and more times (4).

Chronic obstructive pulmonary disease

Chronic obstructive pulmonary disease was defined as using claims of ICD-10 codes (J42, J43 (except J43.0), J44) with more than two times of admission or outpatient department (5).

Renal disease

Renal disease was defined as using claims of ICD–10 codes (N17-19, I12-13, E08.2, E10.2, E11.2, E13.2) more than two times or estimated glomerular filtration rate (eGFR) of <60 $mL/min/1.73m^2$ (6).

Liver disease

Liver disease was defined as using claims of ICD-10 codes (B18, K70, K71, K72, K73, K74, K76.1) with more than two times of admission or outpatient department (7).

Cancer

Cancer was defined as using claims of ICD-10 code(C00–C97) more than two times with cancer-specific deductible code (V027, V193-4) from the Health Insurance Review and Assessment Service (8).

Charlson comorbidity index (0, 1, or ≥2).

The Charlson comorbidity index score was calculated for each subject based on diseases diagnosed before index date and divided into three groups (0, 1, and ≥2 scores) (9).

Social determinants of health (SDoH)

Social determinants of health including depressive symptoms, anxiety symptoms, or stress symptoms was defined as using claims of ICD-10 codes and low-income status. First, low-income status defined as individuals in the lowest 0–4 deciles of income. Second, diagnosis of depression or anxiety/stress-related disorders identified using the following ICD-10 codes; Depression: F32, F33 and Anxiety-related and stress-related disorders: F40–F48 (10, 11).

Inflammatory bowel disease

Inflammatory bowel disease was defined as using claims of ICD-10 code (K50, K51) more than two times. ICD-10 K50 code was used to identify patients with Crohn’s disease, whereas the K51 codes was used to identify patients with ulcerative colitis (12).

Supplementary Method 2

Propensity scores were estimated using a multivariate logistic regression model including demographic, lifestyle, and clinical variables (age, sex, body mass index [BMI], household income, smoking status, alcohol consumption, regular physical activity, hypertension, diabetes mellitus, dyslipidemia, stroke, myocardial infarction, chronic obstructive pulmonary disease [COPD], renal disease, liver disease, cancer, and Charlson comorbidity index [CCI]). We performed matching at a 1:5 ratio (one patient with hemorrhoidal disease matched to five controls). This ratio was selected because statistical power increases substantially up to about 1:4 or 1:5, with minimal additional gain beyond this point (13). Thus, we considered 1:5 matching to provide an optimal balance between bias reduction and efficiency. The matching was implemented in SAS using the OneToManyMTCH macro, which is based on the greedy nearest-neighbor technique described (14). In this method, cases are matched to controls by progressively relaxing the precision of the propensity score from eight to one decimal place. This can be regarded as a variant of caliper matching, where the caliper is defined by decimal digits rather than a fixed numerical distance. Covariate balance was assessed using standardized mean differences, with values below 0.1 indicating adequate balance.

Supplementary Method 3

Calculation of a standardized mean difference on a categorical variable

For categorical variables, we converted them into binary format by coding the category of interest as 1 and all others as 0. In this context, the group-specific means represent the proportion of individuals in each group who belong to the category of interest. That is, the mean corresponds to the proportion. The Standardized Mean Difference (SMD) was then calculated using the following formula:

$$\mathrm{SMD} = \frac{p_1 - p_0}{\sqrt{\frac{p_1(1-p_1) + p_0(1-p_0)}{2}}}$$

where p_1 is the proportion of individuals with the category of interest in the hemorrhoidal disease (+) group, and p_0 is the proportion in the hemorrhoidal disease (-) group. This method is widely used and statistically appropriate for evaluating balance between two groups when dealing with binary (categorical) variables. We have applied this approach consistently throughout the analysis.

Supplementary Method 4

Mediation analysis on the association between hemorrhoidal disease and the incidence risk of dementia, considering SDoH as a mediator

We conducted a mediation analysis based on the Cox proportional hazards model to evaluate whether the effect of hemorrhoidal disease on dementia is mediated through a selected Social Determinant of Health (SDoH) variable. Our approach consisted of the following steps:

Step1. We first estimated the effect of hemorrhoidal disease on the mediator (SDoH) using a logistic regression model. The predicted values from this model were used to compute mediation weights.

Step2. Using these weights, we then fitted a weighted Cox proportional hazards model to estimate the direct and indirect effects of hemorrhoidal disease on dementia. Specifically, the natural direct effect reflects the effect of hemorrhoidal disease on dementia not through the mediator, and the natural indirect effect represents the indirect pathway through the mediator. This approach follows the counterfactual-based Natural Effect Model framework, which is increasingly applied in causal mediation analysis. We implemented this framework in R using the mets and medflex packages, both of which support mediation analysis in the context of survival outcomes (15).

Reference

1. Lee KH, Lee CM, Kwon HT, Oh S-W. Relationship between Obesity and Smoking in Korean Men: Data Analyses from the Third and Fourth Korea National Health and Nutrition Examination Surveys (Knhanes). *JKSRNT* (2010) 1(2):115-23. Epub 07/15. doi: 10.25055/JKSRNT.2010.1.2.115.

2. Yang JH, Choi CK, Kim HY, Heo YR, Shin MH. Association between Alcohol Drinking Status and Depressive Symptoms in Korean Adults. *Chonnam medical journal* (2021) 57(1):68-75. Epub 2021/02/05. doi: 10.4068/cmj.2021.57.1.68.

3. Chun MY. Validity and Reliability of Korean Version of International Physical Activity Questionnaire Short Form in the Elderly. *Korean J Fam Med* (2012) 33(3):144-51. Epub 2012/07/13. doi: 10.4082/kjfm.2012.33.3.144.

4. Choi E-K. Cardiovascular Research Using the Korean National Health Information Database. *Korean Circ J* (2020) 50(9):754-72.

5. Park SC, Kim YS, Kang YA, Park EC, Shin CS, Kim DW, et al. Hemoglobin and Mortality in Patients with Copd: A Nationwide Population-Based Cohort Study. *International journal of chronic obstructive pulmonary disease* (2018) 13:1599-605. Epub 2018/05/29. doi: 10.2147/copd.s159249.

6. Kim J, Jeon J, Lee HS, Lee KY. Association between the Risk for Cardiovascular Events and Antiviral Treatment for Herpes Zoster. *Clinical infectious diseases : an official publication of the Infectious Diseases Society of America* (2021) 73(5):758-64. Epub 2020/09/15. doi: 10.1093/cid/ciaa1384.

7. Naghavi M, Abajobir AA, Abbafati C, Abbas KM, Abd-Allah F, Abera SF, et al. Global, Regional, and National Age-Sex Specific Mortality for 264 Causes of Death, 1980–2016: A Systematic Analysis for the Global Burden of Disease Study 2016. *The Lancet* (2017) 390(10100):1151-210. doi: 10.1016/S0140-6736(17)32152-9.

8. Lim H, Lee Y-H, Bae S, Koh D-H, Yoon M, Lee B-E, et al. Cancer Cluster among Small Village Residents near the Fertilizer Plant in Korea. *PLOS ONE* (2021) 16(2):e0247661. doi: 10.1371/journal.pone.0247661.

9. Kwon HS, Suh J, Kim MH, Yoo B, Han M, Koh IS, et al. Five-Year Community Management Rate for Dementia Patients: A Proposed Indicator for Dementia Policies. *Journal of clinical neurology (Seoul, Korea)* (2022) 18(1):24-32. Epub 2022/01/13. doi: 10.3988/jcn.2022.18.1.24.

10. Ridley M, Rao G, Schilbach F, Patel V. Poverty, Depression, and Anxiety: Causal Evidence and Mechanisms. *Science* (2020) 370(6522):eaay0214. doi: doi:10.1126/science.aay0214.

11. Wilkinson LL, Long-Daniels A, Appah M, Zhai Y, Watson DM, Walker K, et al. The Association between Social Determinants of Health and Depressive Disorders: A 2017 Behavioral Risk Factor Surveillance System (Brfss) Analysis. *Psychiatry International* (2023) 4(2):147-59.

12. Shrestha S, Olén O, Eriksson C, Everhov Å H, Myrelid P, Visuri I, et al. The Use of Icd Codes to Identify Ibd Subtypes and Phenotypes of the Montreal Classification in the Swedish National Patient Register. *Scand J Gastroenterol* (2020) 55(4):430-5. Epub 2020/05/07. doi: 10.1080/00365521.2020.1740778.

13. Woodward M. Epidemiology: study design and data analysis: CRC press; 2013.

14. Parsons LS, editor Reducing Bias in a Propensity Score Matched-Pair Sample Using Greedy Matching Techniques2001.

15. Steen J, Loeys T, Moerkerke B, Vansteelandt S. Medflex: An R Package for Flexible Mediation Analysis Using Natural Effect Models. *Journal of Statistical Software* (2017) 76(11):1 - 46. doi: 10.18637/jss.v076.i11.
