# Supplementary material for: Association of hemorrhoidal disease with dementia risk: a nationwide cohort study
Source: Front Neurol. 2025 Oct 2;16:1655944. doi: 10.3389/fneur.2025.1655944 (PMC12527892; doi:10.3389/fneur.2025.1655944)
Supplement: Supplementary file 3 [file Table_1.DOCX]

**Supplementary Table 1.** Results of Cox regression analysis for the association of hemorrhoidal disease with incidence risk of all-cause dementia.

| Variables | Before PSM N=358,139 | After 1:5 PSM N=138,792 |
| --- | --- | --- |
|  | Adjusted  HR (95%CI) | Adjusted  HR (95%CI) |
| Without hemorrhoidal disease | Reference | Reference |
| With hemorrhoidal disease | 1.228 (1.194-1.263) | 1.243 (1.199-1.288) |
| Age, years | 1.102 (1.101-1.103) | 1.110 (1.108-1.113) |
| Sex |  |  |
| Male | Reference | Reference |
| Female | 1.425 (1.399-1.451) | 1.486 (1.432-1.542) |
| Body mass index (kg/m^2^) | 1.001 (0.998-1.003) | 1.006 (1.001-1.012) |
| Household income |  |  |
| Low | Reference | Reference |
| Middle | 1.003 (0.986-1.020) | 0.983 (0.947-1.020) |
| High | 0.889 (0.873-0.905) | 0.840 (0.808-0.873) |
| Smoking status |  |  |
| Never | Reference | Reference |
| Former | 0.969 (0.939-1.000) | 1.004 (0.948-1.064) |
| Current | 1.071 (1.047-1.096) | 1.066 (1.016-1.119) |
| Alcohol consumption (days/week) |  |  |
| None | Reference | Reference |
| 1-2 times | 0.939 (0.921-0.958) | 0.957 (0.920-0.995) |
| 3-4 times | 1.013 (0.978-1.049) | 1.018 (0.949-1.092) |
| ≥ 5 times | 1.151 (1.111-1.192) | 1.165 (1.073-1.264) |
| Regular physical activity (days/week) |  |  |
| None | Reference | Reference |
| 1-4 days | 0.866 (0.852-0.882) | 0.865 (0.836-0.895) |
| ≥ 5 days | 0.924 (0.903-0.945) | 0.904 (0.862-0.948) |
| Comorbidities |  |  |
| Hypertension | 1.072 (1.055-1.089) | 1.079 (1.042-1.118) |
| Diabetes mellitus | 1.243 (1.218-1.269) | 1.249 (1.186-1.315) |
| Dyslipidemia | 1.119 (1.098-1.141) | 1.145 (1.103-1.189) |
| Stroke | 1.575 (1.416-1.753) | 1.705 (1.358-2.141) |
| Myocardial Infarction | 1.127 (0.938-1.354) | 1.168 (0.800-1.703) |
| COPD | 1.180 (1.160-1.200) | 1.205 (1.165-1.246) |
| Renal disease | 1.075 (1.032-1.119) | 1.038 (0.955-1.128) |
| Liver disease | 1.226 (1.201-1.252) | 1.253 (1.205-1.302) |
| Cancer | 1.146 (1.105-1.188) | 1.125 (1.050-1.206) |
| SDoH | 1.456 (1.390-1.525) | 1.593 (1.430-1.775) |
| IBD | 1.140 (1.063-1.222) | 1.170 (1.017-1.347) |
| Charlson comorbidity index |  |  |
| 0 | Reference | Reference |
| 1 | 1.062 (1.034-1.092) | 1.111 (1.048-1.178) |
| 2 or more | 1.014 (0.936-1.099) | 0.906 (0.751-1.091) |

PSM, propensity score matching; N, number; HR, hazard ratio; CI, confidence interval; COPD, chronic obstructive pulmonary disease; SDoH, social determinants of health; IBD, inflammatory bowel disease.

**Supplementary Table 2.** Results of Wald Chi-square tests from the Cox proportional hazards model before propensity score matching.

|  |  |  | All-cause dementia | | Alzheimer’s disease | | Vascular dementia | |
| --- | --- | --- | --- | --- | --- | --- | --- | --- |
| Variables | Test Type | DF | Wald χ² | P-value | Wald χ² | P-value | Wald χ² | P-value |
| Unadjusted Model |  |  |  |  |  |  |  |  |
| Overall Model | Global | 1 | 56.800 | <0.001 | 35.908 | <.001 | 12.264 | <0.001 |
| Adjusted Model |  |  |  |  |  |  |  |  |
| Overall Model | Global | 26 | 79669.454 | <0.001 | 57133.027 | <.001 | 16841.816 | <0.001 |
| With hemorrhoidal disease | Type3 | 1 | 212.315 | <0.001 | 11.849 | <.001 | 98.110 | <0.001 |
| Age, years | Type3 | 1 | 43968.580 | <0.001 | 22043.465 | <.001 | 12108.181 | <0.001 |
| Sex | Type3 | 1 | 1432.626 | <0.001 | 538.983 | <.001 | 24.654 | <0.001 |
| Body mass index (kg/m^2^) | Type3 | 1 | 0.426 | 0.514 | 15.228 | <.001 | 7.716 | 0.005 |
| Household income | Type3 | 2 | 219.315 | <0.001 | 93.770 | <.001 | 49.271 | <0.001 |
| Smoking status | Type3 | 2 | 48.655 | <0.001 | 68.553 | <.001 | 43.952 | <0.001 |
| Alcohol consumption (days/week) | Type3 | 3 | 130.838 | <0.001 | 71.370 | <.001 | 18.457 | <0.001 |
| Regular physical activity (days/week) | Type3 | 2 | 273.117 | <0.001 | 169.120 | <.001 | 77.292 | <0.001 |
| Hypertension | Type3 | 1 | 76.029 | <0.001 | 28.158 | <.001 | 139.206 | <0.001 |
| Diabetes mellitus | Type3 | 1 | 436.357 | <0.001 | 468.546 | <.001 | 184.305 | <0.001 |
| Dyslipidemia | Type3 | 1 | 129.822 | <0.001 | 46.158 | <.001 | 38.252 | <0.001 |
| Stroke | Type3 | 1 | 69.993 | <0.001 | 37.148 | <.001 | 47.011 | <0.001 |
| Myocardial Infarction | Type3 | 1 | 1.637 | 0.200 | 1.710 | 0.191 | 0.265 | 0.606 |
| COPD | Type3 | 1 | 381.341 | <0.001 | 135.149 | <.001 | 12.363 | <0.001 |
| Renal disease | Type3 | 1 | 12.139 | <0.001 | 14.859 | <.001 | 19.563 | <0.001 |
| Liver disease | Type3 | 1 | 376.759 | <0.001 | 112.846 | <.001 | 25.116 | <0.001 |
| Cancer | Type3 | 1 | 54.536 | <0.001 | 27.691 | <.001 | 12.963 | <0.001 |
| SDoH | Type3 | 1 | 254.006 | <0.001 | 100.621 | <.001 | 51.898 | <0.001 |
| IBD | Type3 | 1 | 13.540 | <0.001 | 4.338 | 0.037 | 1.278 | 0.258 |
| Charlson comorbidity index | Type3 | 2 | 18.812 | <0.001 | 7.819 | 0.020 | 8.288 | 0.015 |

DF, degree of freedom; COPD, chronic obstructive pulmonary disease; SDoH, social determinants of health; IBD, inflammatory bowel disease.

S2 Table presents the Wald Chi-square test results from the Cox proportional hazards model applied to the cohort before propensity score matching (N = 358,139). The global test (DF = 26) confirms overall model significance, while Type 3 tests show the independent effects of each covariate. The chi-square statistic (χ²) reflects the strength of association with the hazard.

**Supplementary Table 3.** Results of Wald Chi-square tests from the Cox proportional hazards model after propensity score matching.

|  |  |  | All-cause dementia | | Alzheimer’s disease | | Vascular dementia | |
| --- | --- | --- | --- | --- | --- | --- | --- | --- |
| Variables | Test Type | DF | Wald χ² | P-value | Wald χ² | P-value | Wald χ² | P-value |
| Unadjusted Model |  |  |  |  |  |  |  |  |
| Overall Model | Global | 1 | 117.657 | <0.001 | 2.399 | 0.121 | 1.410 | 0.235 |
| Adjusted Model |  |  |  |  |  |  |  |  |
| Overall Model | Global | 26 | 32255.940 | <0.001 | 22102.189 | <.001 | 6655.687 | <0.001 |
| With hemorrhoidal disease | Type3 | 1 | 139.945 | <0.001 | 5.509 | 0.018 | 6.651 | 0.009 |
| Age, years | Type3 | 1 | 12863.742 | <0.001 | 6997.856 | <.001 | 2464.504 | <0.001 |
| Sex | Type3 | 1 | 439.849 | <0.001 | 128.794 | <.001 | 1.718 | 0.190 |
| Body mass index (kg/m^2^) | Type3 | 1 | 5.539 | 0.018 | 1.481 | 0.223 | 10.756 | 0.001 |
| Household income | Type3 | 2 | 131.460 | <0.001 | 70.669 | <.001 | 38.336 | <0.001 |
| Smoking status | Type3 | 2 | 9.498 | 0.008 | 13.311 | 0.001 | 19.132 | <0.001 |
| Alcohol consumption (days/week) | Type3 | 3 | 34.156 | <0.001 | 36.047 | <.001 | 10.823 | 0.012 |
| Regular physical activity (days/week) | Type3 | 2 | 125.840 | <0.001 | 71.734 | <.001 | 38.650 | <0.001 |
| Hypertension | Type3 | 1 | 18.241 | <0.001 | 11.158 | <.001 | 29.246 | <0.001 |
| Diabetes mellitus | Type3 | 1 | 70.894 | <0.001 | 41.831 | <.001 | 52.787 | <0.001 |
| Dyslipidemia | Type3 | 1 | 49.144 | <0.001 | 7.612 | 0.005 | 4.397 | 0.036 |
| Stroke | Type3 | 1 | 21.121 | <0.001 | 12.091 | <.001 | 16.763 | <0.001 |
| Myocardial Infarction | Type3 | 1 | 0.647 | 0.421 | 1.294 | 0.255 | 0.755 | 0.384 |
| COPD | Type3 | 1 | 117.218 | <0.001 | 28.418 | <.001 | 9.934 | 0.001 |
| Renal disease | Type3 | 1 | 0.769 | 0.380 | 0.667 | 0.414 | 6.707 | 0.009 |
| Liver disease | Type3 | 1 | 128.073 | <0.001 | 28.832 | <.001 | 12.114 | <0.001 |
| Cancer | Type3 | 1 | 11.196 | <0.001 | 8.810 | 0.003 | 6.940 | 0.008 |
| SDoH | Type3 | 1 | 71.475 | <0.001 | 20.881 | 0.048 | 12.493 | <0.001 |
| IBD | Type3 | 1 | 4.821 | 0.028 | 1.009 | 0.315 | 1.182 | 0.276 |
| Charlson comorbidity index | Type3 | 2 | 11.252 | 0.003 | 0.762 | 0.683 | 0.198 | 0.905 |

DF, degree of freedom; COPD, chronic obstructive pulmonary disease; SDoH, social determinants of health; IBD, inflammatory bowel disease.

S3 Table presents the Wald Chi-square test results from the Cox proportional hazards model applied to the cohort after propensity score matching (N = 138,792). The global test (DF = 26) confirms overall model significance, while Type 3 tests show the independent effects of each covariate. The chi-square statistic (χ²) reflects the strength of association with the hazard.

**Supplementary Table 4.** Results of Cox regression analysis for the association of hemorrhoidal disease with incidence risk of Alzheimer’s disease.

| Variables | Before PSM N=358,139 | After 1:5 PSM N=138,792 |
| --- | --- | --- |
|  | Adjusted  HR (95%CI) | Adjusted  HR (95%CI) |
| Without hemorrhoidal disease | Reference | Reference |
| With hemorrhoidal disease | 1.073 (1.028-1.121) | 1.075 (1.012-1.142) |
| Age, years | 1.142 (1.140-1.143) | 1.159 (1.155-1.163) |
| Sex |  |  |
| Male | Reference | Reference |
| Female | 1.367 (1.331-1.403) | 1.418 (1.335-1.506) |
| Body mass index (kg/m^2^) | 0.994 (0.991-0.998) | 0.995 (0.987-1.003) |
| Household income |  |  |
| Low | Reference | Reference |
| Middle | 0.989 (0.965-1.014) | 0.983 (0.925-1.044) |
| High | 0.889 (0.866-0.912) | 0.811 (0.762-0.864) |
| Smoking status |  |  |
| Never | Reference | Reference |
| Former | 0.991 (0.946-1.038) | 1.050 (0.954-1.156) |
| Current | 1.142 (1.105-1.180) | 1.188 (1.096-1.287) |
| Alcohol consumption (days/week) |  |  |
| None | Reference | Reference |
| 1-2 times | 0.924 (0.897-0.953) | 0.927 (0.868-0.990) |
| 3-4 times | 0.989 (0.938-1.042) | 0.959 (0.852-1.079) |
| ≥ 5 times | 1.143 (1.089-1.200) | 1.250 (1.101-1.419) |
| Regular physical activity (days/week) |  |  |
| None | Reference | Reference |
| 1-4 days | 0.839 (0.817-0.861) | 0.792 (0.749-0.839) |
| ≥ 5 days | 0.934 (0.905-0.965) | 0.883 (0.819-0.951) |
| Comorbidities |  |  |
| Hypertension | 1.061 (1.038-1.085) | 1.097 (1.039-1.159) |
| Diabetes mellitus | 1.359 (1.322-1.397) | 1.299 (1.200-1.407) |
| Dyslipidemia | 1.093 (1.064-1.124) | 1.089 (1.025-1.157) |
| Stroke | 1.536 (1.338-1.763) | 1.802 (1.293-2.511) |
| Myocardial Infarction | 1.175 (0.923-1.497) | 1.405 (0.782-2.524) |
| COPD | 1.149 (1.122-1.176) | 1.156 (1.096-1.219) |
| Renal disease | 1.113 (1.054-1.176) | 1.054 (0.929-1.196) |
| Liver disease | 1.175 (1.140-1.210) | 1.187 (1.115-1.262) |
| Cancer | 1.142 (1.087-1.200) | 1.174 (1.056-1.304) |
| SDoH | 1.387 (1.301-1.479) | 1.476 (1.249-1.743) |
| IBD | 1.110 (1.006-1.225) | 1.121 (0.897-1.401) |
| Charlson comorbidity index |  |  |
| 0 | Reference | Reference |
| 1 | 1.043 (1.002-1.084) | 1.006 (0.915-1.105) |
| 2 or more | 1.111 (0.999-1.236) | 1.122 (0.843-1.495) |

PSM, propensity score matching; N, number; HR, hazard ratio; CI, confidence interval; COPD, chronic obstructive pulmonary disease; SDoH, social determinants of health; IBD, inflammatory bowel disease.

**Supplementary Table 5.** Results of Cox regression analysis for the association of hemorrhoidal disease with incidence risk of vascular dementia.

| Variables | Before PSM  N=358,139 | After 1:5 PSM N=138,792 |
| --- | --- | --- |
|  | Adjusted  HR (95%CI) | Adjusted  HR (95%CI) |
| Without hemorrhoidal disease | Reference | Reference |
| With hemorrhoidal disease | 1.130 (1.103-1.157) | 1.086 (1.020-1.152) |
| Age, years | 1.110 (1.108-1.112) | 1.120 (1.115-1.125) |
| Sex |  |  |
| Male | Reference | Reference |
| Female | 1.110 (1.065-1.157) | 1.059 (0.972-1.154) |
| Body mass index (kg/m^2^) | 1.008 (1.002-1.013) | 1.020 (1.008-1.033) |
| Household income |  |  |
| Low | Reference | Reference |
| Middle | 1.022 (0.983-1.063) | 0.981 (0.899-1.071) |
| High | 0.890 (0.853-0.928) | 0.814 (0.744-0.891) |
| Smoking status |  |  |
| Never | Reference | Reference |
| Former | 0.969 (0.903-1.040) | 0.967 (0.847-1.105) |
| Current | 1.168 (1.111-1.228) | 1.230 (1.103-1.373) |
| Alcohol consumption (days/week) |  |  |
| None | Reference | Reference |
| 1-2 times | 0.943 (0.901-0.988) | 0.938 (0.855-1.030) |
| 3-4 times | 1.017 (0.940-1.100) | 0.926 (0.785-1.092) |
| ≥ 5 times | 1.113 (1.032-1.201) | 1.219 (1.022-1.453) |
| Regular physical activity (days/week) |  |  |
| None | Reference | Reference |
| 1-4 days | 0.830 (0.796-0.865) | 0.781 (0.720-0.847) |
| ≥ 5 days | 0.945 (0.898-0.995) | 0.956 (0.859-1.065) |
| Comorbidities |  |  |
| Hypertension | 1.231 (1.189-1.274) | 1.240 (1.147-1.341) |
| Diabetes mellitus | 1.350 (1.293-1.410) | 1.519 (1.357-1.699) |
| Dyslipidemia | 1.147 (1.099-1.198) | 1.096 (1.006-1.194) |
| Stroke | 1.934 (1.602-2.335) | 2.574 (1.637-4.048) |
| Myocardial Infarction | 1.103 (0.760-1.599) | 1.436 (0.635-3.247) |
| COPD | 1.070 (1.030-1.111) | 1.132 (1.048-1.222) |
| Renal disease | 1.204 (1.109-1.307) | 1.256 (1.057-1.493) |
| Liver disease | 1.127 (1.075-1.180) | 1.170 (1.071-1.278) |
| Cancer | 1.153 (1.067-1.246) | 1.219 (1.052-1.412) |
| SDoH | 1.442 (1.305-1.593) | 1.517 (1.204-1.910) |
| IBD | 1.095 (0.936-1.281) | 1.186 (0.872-1.615) |
| Charlson comorbidity index |  |  |
| 0 | Reference | Reference |
| 1 | 1.068 (1.004-1.135) | 0.963 (0.840-1.104) |
| 2 or more | 1.186 (1.009-1.394) | 0.956 (0.644-1.419) |

PSM, propensity score matching; N, number; HR, hazard ratio; CI, confidence interval; COPD, chronic obstructive pulmonary disease; SDoH, social determinants of health; IBD, inflammatory bowel disease.

**Supplementary Table 6.** Subgroup analysis of hazard ratios with 95% confidence intervals and Wald chi-square statistics from the Cox model before propensity score matching.

| Subgroup | | HR (95% CI) | DF | Wald χ² | P-value |
| --- | --- | --- | --- | --- | --- |
| Age | <65 year | 1.206 (1.166-1.248) | 1 | 116.719 | <0.001 |
|  | ≥65 year | 1.149 (1.097-1.204) | 1 | 34.222 | <0.001 |
| Sex | Female | 1.047 (1.008-1.089) | 1 | 5.426 | 0.019 |
|  | Male | 1.271 (1.222-1.322) | 1 | 142.827 | <0.001 |
| BMI | < 25 kg/m^2^ | 1.089 (1.052-1.128) | 1 | 22.958 | <0.001 |
|  | ≥ 25 kg/m^2^ | 1.152 (1.100-1.205) | 1 | 37.016 | <0.001 |
| Household income | Low | 1.140 (1.103-1.179) | 1 | 59.419 | <0.001 |
|  | High | 1.106 (1.054-1.161) | 1 | 16.684 | <0.001 |
| Current smoker | No | 1.076 (1.044-1.108) | 1 | 23.292 | <0.001 |
|  | Yes | 1.194 (1.110-1.283) | 1 | 23.028 | <0.001 |
| Heavy drinker | No | 1.103 (1.072-1.136) | 1 | 43.920 | <0.001 |
|  | Yes | 1.175 (1.074-1.286) | 1 | 12.315 | <0.001 |
| Regular exercise | No | 1.084 (1.044-1.124) | 1 | 18.338 | <0.001 |
|  | Yes | 1.225 (1.175-1.277) | 1 | 91.325 | <0.001 |
| Hypertension | No | 1.130 (1.098-1.164) | 1 | 67.364 | <0.001 |
|  | Yes | 1.144 (1.054-1.243) | 1 | 10.223 | 0.001 |
| Diabetes mellitus | No | 1.132 (1.095-1.169) | 1 | 55.238 | <0.001 |
|  | Yes | 1.131 (1.075-1.190) | 1 | 22.544 | <0.001 |
| Dyslipidemia | No | 1.037 (1.004-1.072) | 1 | 4.723 | 0.029 |
|  | Yes | 1.096 (1.042-1.153) | 1 | 12.601 | <0.001 |
| Stroke | No | 1.109 (1.078-1.140) | 1 | 52.597 | <0.001 |
|  | Yes | 1.183 (0.845-1.655) | 1 | 0.960 | 0.327 |
| Myocardial Infarction | No | 1.112 (1.081-1.143) | 1 | 55.680 | <0.001 |
|  | Yes | 0.668 (0.294-1.520) | 1 | 0.927 | 0.335 |
| COPD | No | 1.063 (1.028-1.100) | 1 | 12.516 | <0.001 |
|  | Yes | 1.026 (0.978-1.076) | 1 | 1.110 | 0.292 |
| Renal disease | No | 1.104 (1.074-1.136) | 1 | 47.756 | <0.001 |
|  | Yes | 1.106 (0.968-1.265) | 1 | 2.178 | 0.140 |
| Liver disease | No | 1.044 (1.011-1.078) | 1 | 6.920 | 0.008 |
|  | Yes | 1.112 (1.054-1.174) | 1 | 14.896 | <0.001 |
| Cancer | No | 1.099 (1.068-1.131) | 1 | 41.686 | <0.001 |
|  | Yes | 1.060 (0.951-1.182) | 1 | 1.103 | 0.293 |
| SDoH | No | 1.097 (1.066-1.128) | 1 | 41.210 | <0.001 |
|  | Yes | 1.194 (1.042-1.369) | 1 | 6.485 | 0.010 |
| IBD | No | 1.103 (1.072-1.134) | 1 | 46.716 | <0.001 |
|  | Yes | 1.132 (0.952-1.345) | 1 | 1.978 | 0.159 |
| Charlson comorbidity index | 0 | 1.077 (1.046-1.108) | 1 | 25.500 | <0.001 |
|  | 1 or more | 1.423 (1.297-1.561) | 1 | 55.709 | <0.001 |

HR, hazard ratio; CI, confidence interval; DF, degree of freedom; BMI, body mass index; COPD, chronic obstructive pulmonary disease; SDoH, social determinants of health; IBD, inflammatory bowel disease. Subgroup-specific hazard ratios, 95% confidence intervals, and Wald chi-square statistics from the Cox model are summarized. All results are based on the cohort before propensity score matching (N = 358,139).

**Supplementary Table 7.** Subgroup analysis of hazard ratios with 95% confidence intervals and Wald chi-square statistics from the Cox model after propensity score matching.

| Subgroup | | HR (95% CI) | DF | Wald χ² | P-value |
| --- | --- | --- | --- | --- | --- |
| Age | <65 year | 1.261 (1.212-1.312) | 1 | 131.479 | <0.001 |
|  | ≥65 year | 1.115 (1.037-1.198) | 1 | 8.742 | 0.003 |
| Sex | Female | 1.107 (1.056-1.161) | 1 | 17.671 | <0.001 |
|  | Male | 1.288 (1.229-1.349) | 1 | 113.407 | <0.001 |
| BMI | < 25 kg/m^2^ | 1.178 (1.131-1.227) | 1 | 62.130 | <0.001 |
|  | ≥ 25 kg/m^2^ | 1.206 (1.141-1.275) | 1 | 43.724 | <0.001 |
| Household income | Low | 1.175 (1.129-1.223) | 1 | 62.484 | <0.001 |
|  | High | 1.209 (1.141-1.280) | 1 | 41.887 | <0.001 |
| Current smoker | No | 1.173 (1.133-1.214) | 1 | 82.053 | <0.001 |
|  | Yes | 1.294 (1.187-1.412) | 1 | 33.880 | <0.001 |
| Heavy drinker | No | 1.183 (1.145-1.223) | 1 | 99.923 | <0.001 |
|  | Yes | 1.240 (1.109-1.386) | 1 | 14.303 | <0.001 |
| Regular exercise | No | 1.122 (1.073-1.173) | 1 | 25.645 | <0.001 |
|  | Yes | 1.274 (1.213-1.339) | 1 | 92.265 | <0.001 |
| Hypertension | No | 1.188 (1.149-1.228) | 1 | 103.139 | <0.001 |
|  | Yes | 1.173 (1.053-1.306) | 1 | 8.438 | 0.003 |
| Diabetes mellitus | No | 1.167 (1.124-1.212) | 1 | 64.503 | <0.001 |
|  | Yes | 1.261 (1.181-1.345) | 1 | 48.875 | <0.001 |
| Dyslipidemia | No | 1.167 (1.124-1.212) | 1 | 64.503 | <0.001 |
|  | Yes | 1.261 (1.181-1.345) | 1 | 48.875 | <0.001 |
| Stroke | No | 1.189 (1.152-1.227) | 1 | 115.757 | <0.001 |
|  | Yes | 1.007 (0.593-1.709) | 1 | 0.001 | 0.974 |
| Myocardial Infarction | No | 1.189 (1.152-1.227) | 1 | 115.757 | <0.001 |
|  | Yes | 0.691 (0.257-1.862) | 1 | 0.535 | 0.464 |
| COPD | No | 1.222 (1.176-1.271) | 1 | 102.347 | <0.001 |
|  | Yes | 1.133 (1.066-1.204) | 1 | 16.168 | <0.001 |
| Renal disease | No | 1.185 (1.148-1.224) | 1 | 107.746 | <0.001 |
|  | Yes | 1.273 (1.060-1.528) | 1 | 6.695 | <0.001 |
| Liver disease | No | 1.181 (1.138-1.225) | 1 | 78.364 | <0.001 |
|  | Yes | 1.217 (1.136-1.303) | 1 | 31.505 | <0.001 |
| Cancer | No | 1.197 (1.159-1.237) | 1 | 117.127 | <0.001 |
|  | Yes | 0.997 (0.860-1.156) | 1 | 0.002 | 0.964 |
| SDoH | No | 1.175 (1.137-1.213) | 1 | 95.459 | <0.001 |
|  | Yes | 1.153 (0.943-1.410) | 1 | 1.925 | 0.165 |
| IBD | No | 1.178 (1.141-1.217) | 1 | 99.173 | <0.001 |
|  | Yes | 1.375 (1.073-1.763) | 1 | 6.320 | 0.011 |
| Charlson comorbidity index | 0 | 1.189 (1.150-1.228) | 1 | 106.928 | <0.001 |
|  | 1 or more | 1.188 (1.050-1.344) | 1 | 7.483 | 0.006 |

HR, hazard ratio; CI, confidence interval; DF, degree of freedom; BMI, body mass index; COPD, chronic obstructive pulmonary disease; SDoH, social determinants of health; IBD, inflammatory bowel disease. Subgroup-specific hazard ratios, 95% confidence intervals, and Wald chi-square statistics from the Cox model are summarized. All results are based on the cohort after propensity score matching (N = 138,792).

**Supplementary Table 8**. Comparative analysis according to whether received surgical procedures/treatments for hemorrhoidal disease or not.

|  | Before PSM | | | | After 1:1 PSM | | |
| --- | --- | --- | --- | --- | --- | --- | --- |
| Variable | Total | Hemorrhoidal disease  Trt (-) | Hemorrhoidal disease  Trt (+) | p-value | Hemorrhoidal disease  Trt (-) | Hemorrhoidal disease  Trt (+) | SMD* |
|  |  | Mean ± SD,  N (%) | Mean ± SD,  N (%) |  | Mean ± SD,  N (%) | Mean ± SD,  N (%) |  |
| Number | 24,489 | 9,891 | 14,598 |  | 9,165 | 9,165 |  |
| Age, years | 53.8 ± 8.8 | 55.6 ± 9.5 | 52.5 ± 8.1 | <.001 | 54.8 ± 8.9 | 54.7 ± 8.6 | 0.006 |
| Sex |  |  |  | 0.012 |  |  | 0.006 |
| Female | 14,506 (59.3) | 5,947 (60.1) | 8,559 (58.6) |  | 5,475 (59.74) | 5,449 (59.5) |  |
| Male | 9,983 (40.7) | 3,944 (39.9) | 6,039 (41.4) |  | 3,690 (40.26) | 3,716 (40.5) |  |
| Body mass index (kg/m^2^) | 24.0 ± 2.8 | 24.0 ± 2.9 | 23.9 ± 2.7 | <.001 | 24.0 ± 2.8 | 24.0 ± 2.8 | 0.000 |
| Household income |  |  |  | 0.190 |  |  | 0.004 |
| Low | 6,060 (24.8) | 2,421 (24.5) | 3,639 (24.9) |  | 2,246 (24.5) | 2,230 (24.3) |  |
| Middle | 8,848 (36.1) | 3,532 (35.7) | 5,316 (36.4) |  | 3,327 (36.3) | 3,272 (35.7) |  |
| High | 9,581 (39.1) | 3,938 (39.8) | 5,643 (38.7) |  | 3,592 (39.2) | 3,663 (40.0) |  |
| Smoking status |  |  |  | <.001 |  |  | 0.010 |
| Never | 17,196 (70.2) | 7,149 (72.3) | 10,047 (68.8) |  | 6,575 (71.7) | 6,536 (71.3) |  |
| Former | 2,670 (10.9) | 1,076 (10.9) | 1,594 (10.9) |  | 1,004 (11.0) | 1,039 (11.3) |  |
| Current | 4,623 (18.9) | 1,666 (16.8) | 2,957 (20.3) |  | 1,586 (17.3) | 1,590 (17.4) |  |
| Alcohol consumption  (days/week) |  |  |  | <.001 |  |  | 0.000 |
| None | 14,013 (57.2) | 5,898 (59.6) | 8,115 (55.6) |  | 5,390 (58.8) | 5,391 (58.8) |  |
| 1-2 times | 8,139 (33.2) | 3,036 (30.7) | 5,103 (35.0) |  | 2,894 (31.6) | 2,922 (31.9) |  |
| 3-4 times | 1,540 (6.3) | 605 (6.1) | 935 (6.4) |  | 573 (6.3) | 549 (6.0) |  |
| ≥5 times | 797 (3.3) | 352 (3.6) | 445 (3.1) |  | 308 (3.4) | 303 (3.3) |  |
| Regular physical activity  (days/week) |  |  |  | 0.001 |  |  | 0.013 |
| None | 11,886 (48.5) | 4,877 (49.3) | 7,009 (48.0) |  | 4,471 (48.8) | 4,413 (48.2) |  |
| 1-4 days | 9,997 (40.8) | 3,905 (39.5) | 6,092 (41.7) |  | 3,685 (40.2) | 3,739 (40.8) |  |
| ≥5 days | 2,606 (10.7) | 1,109 (11.2) | 1,497 (10.3) |  | 1,009 (11.0) | 1,013 (11.0) |  |
| Comorbidities |  |  |  |  |  |  |  |
| Hypertension | 5,288 (21.6) | 2,363 (23.9) | 2,925 (20.0) | <.001 | 2,108 (23.0) | 2,074 (22.6) | 0.009 |
| Diabetes mellitus | 1,852 (7.6) | 896 (9.1) | 956 (6.6) | <.001 | 741 (8.1) | 748 (8.2) | -0.003 |
| Dyslipidemia | 5,718 (23.4) | 2,740 (27.7) | 2,978 (20.4) | <.001 | 2,366 (25.8) | 2,435 (26.6) | -0.017 |
| Stroke | 80 (0.3) | 46 (0.5) | 34 (0.2) | 0.002 | 34 (0.4) | 31 (0.3) | 0.005 |
| Myocardial Infarction | 31 (0.1) | 15 (0.6) | 16 (0.1) | 0.364 | 14 (0.2) | 11 (0.1) | 0.008 |
| COPD | 6,447 (26.3) | 3,013 (30.5) | 3,434 (23.5) | <.001 | 2,622 (28.6) | 2,637 (28.8) | -0.004 |
| Renal disease | 705 (2.9) | 386 (3.9) | 319 (2.2) | <.001 | 289 (3.2) | 286 (3.1) | 0.002 |
| Liver disease | 5,375 (22.0) | 2,576 (26.0) | 2,799 (19.2) | <.001 | 2,218 (24.2) | 2,256 (24.6) | -0.010 |
| Cancer | 1,267 (5.2) | 716 (7.2) | 551 (3.8) | <.001 | 509 (5.6) | 506 (5.5) | 0.001 |
| SDoH | 547 (2.2) | 279 (2.8) | 268 (1.8) | <.001 | 243 (2.6) | 176 (1.9) | 0.044 |
| IBD | 535 (2.2) | 284 (2.9) | 251 (1.7) | <.001 | 258 (2.8) | 178 (1.9) | 0.055 |
| Charlson comorbidity index |  |  |  | 0.019 |  |  | -0.002 |
| 0 | 22,486 (93.2) | 9,078 (92.7) | 13,408 (93.6) |  | 8,515 (92.9) | 8,520 (93.0) |  |
| 1 | 1,507 (6.3) | 659 (6.7) | 848 (5.9) |  | 593 (6.5) | 588 (6.4) |  |
| ≥2 | 134 (0.55) | 61 (0.6) | 73 (0.5) |  | 57 (0.6) | 57 (0.6) |  |

PSM, propensity score matching; Trt, treatment; SD, standard deviation; N, number; SMD, standardized mean difference; COPD, chronic obstructive pulmonary disease; SDoH, social determinants of health; IBD, inflammatory bowel disease. * All standardized mean difference values were <0.1 in the propensity score matched cohort.

**Supplementary Table 9.** Frequency table of surgical procedure/treatment codes for hemorrhoidal disease therapy.

| Hemorrhoidal disease treatment group, N=14,598 | | | | | | | |
| --- | --- | --- | --- | --- | --- | --- | --- |
| Number of treatment = 1 | | Number of treatment = 2 | | Number of treatment = 3 | | Number of treatment = 4 | |
| Procedure code | N (%) | Procedure code | N (%) | Procedure code | N (%) | Procedure code | N (%) |
| Q3012 | 862 (5.9%) | Q3012 & Q3013 | 302 (2.2%) | Q3012 & Q3013 & Q3014 | 0 (0.0%) | Q3014 & Q3015 & Q3016 & Q3017 | 0 (0.0%) |
| Q3013 | 11,591 (79.4%) | Q3012 & Q3014 | 1 (0.0%) | Q3012 & Q3013 & Q3015 | 15 (0.1%) | Q3013 & Q3015 & Q3016 & Q3017 | 0 (0.0%) |
| Q3014 | 208 (1.4%) | Q3012 & Q3015 | 26 (0.2%) | Q3012 & Q3013 & Q3016 | 7 (0.0%) | Q3013 & Q3014 & Q3016 & Q3017 | 0 (0.0%) |
| Q3015 | 258 (1.8%) | Q3012 & Q3016 | 21 (0.1%) | Q3012 & Q3013 & Q3017 | 11 (0.1%) | Q3013 & Q3014 & Q3015 & Q3017 | 0 (0.0%) |
| Q3016 | 239 (1.7%) | Q3012 & Q3017 | 10 (0.1%) | Q3012 & Q3014 & Q3015 | 0 (0.0%) | Q3013 & Q3014 & Q3015 & Q3016 | 0 (0.0%) |
| Q3017 | 202 (1.4%) | Q3013 & Q3014 | 19 (0.1%) | Q3012 & Q3014 & Q3016 | 0 (0.0%) | Q3012 & Q3015 & Q3016 & Q3017 | 0 (0.0%) |
|  |  | Q3013 & Q3015 | 518 (3.5%) | Q3012 & Q3014 & Q3017 | 0 (0.0%) | Q3012 & Q3014 & Q3016 & Q3017 | 0 (0.0%) |
|  |  | Q3013 & Q3016 | 167 (1.1%) | Q3012 & Q3015 & Q3016 | 1 (0.0%) | Q3012 & Q3014 & Q3015 & Q3017 | 0 (0.0%) |
|  |  | Q3013 & Q3017 | 75 (0.5%) | Q3012 & Q3015 & Q3017 | 0 (0.0%) | Q3012 & Q3014 & Q3015 & Q3016 | 0 (0.0%) |
|  |  | Q3014 & Q3015 | 9 (0.1%) | Q3012 & Q3016 & Q3017 | 2 (0.0%) | Q3012 & Q3013 & Q3016 & Q3017 | 2 (0.0%) |
|  |  | Q3014 & Q3016 | 2 (0.0%) | Q3013 & Q3014 & Q3015 | 3 (0.0%) | Q3012 & Q3013 & Q3015 & Q3017 | 0 (0.0%) |
|  |  | Q3014 & Q3017 | 0 (0.0%) | Q3013 & Q3014 & Q3016 | 0 (0.0%) | Q3012 & Q3013 & Q3015 & Q3016 | 2 (0.0%) |
|  |  | Q3015 & Q3016 | 9 (0.1%) | Q3013 & Q3014 & Q3017 | 0 (0.0%) | Q3012 & Q3013 & Q3014 & Q3017 | 0 (0.0%) |
|  |  | Q3015 & Q3017 | 8 (0.1%) | Q3013 & Q3015 & Q3016 | 10 (0.1%) | Q3012 & Q3013 & Q3014 & Q3016 | 0 (0.0%) |
|  |  | Q3016 & Q3017 | 6 (0.0%) | Q3013 & Q3015 & Q3017 | 6 (0.0%) | Q3012 & Q3013 & Q3014 & Q3015 | 0 (0.0%) |
|  |  |  |  | Q3013 & Q3016 & Q3017 | 2 (0.0%) |  |  |
|  |  |  |  | Q3014 & Q3015 & Q3016 | 2 (0.0%) |  |  |
|  |  |  |  | Q3014 & Q3015 & Q3017 | 1 (0.0%) |  |  |
|  |  |  |  | Q3014 & Q3016 & Q3017 | 1 (0.0%) |  |  |
|  |  |  |  | Q3015 & Q3016 & Q3017 | 0 (0.0%) |  |  |

N, number.

The procedure code names of the treatment corresponding to each code are as follows: thrombectomy and excision of the skin tag (Q3015)**;** surgery for strangulated circumferential hemorrhoids (Q3014); thrombosed hemorrhoid surgery (Q3012); coagulation, cauterization, sclerotherapy, and rubber band ligation (Q3016)**;** circular stapled hemorrhoidectomy (Q3017)**;** hemorrhoidectomy (Q3013).

**Supplementary Table 10.** Results of Cox regression analysis for the association of surgical procedure/treatment for hemorrhoidal disease with incidence risk of all-cause dementia.

| Variables | Before PSM N=24,489 | After 1:1 PSM N=18,330 |
| --- | --- | --- |
|  | Adjusted  HR (95%CI) | Adjusted  HR (95%CI) |
| Without treatment | Reference | Reference |
| With treatment | 0.904 (0.856-0.954) | 0.925 (0.872-0.981) |
| Age, years | 1.100 (1.096-1.103) | 1.098 (1.094-1.102) |
| Sex |  |  |
| Male | Reference | Reference |
| Female | 1.436 (1.345-1.534) | 1.420 (1.321-1.526) |
| Body mass index (kg/m^2^) | 1.006 (0.996-1.015) | 1.004 (0.993-1.014) |
| Household income |  |  |
| Low | Reference | Reference |
| Middle | 0.946 (0.883-1.013) | 0.919 (0.852-0.991) |
| High | 0.834 (0.777-0.895) | 0.831 (0.768-0.898) |
| Smoking status |  |  |
| Never | Reference | Reference |
| Former | 0.999 (0.901-1.108) | 1.012 (0.904-1.133) |
| Current | 1.052 (0.965-1.148) | 1.052 (0.955-1.159) |
| Alcohol consumption (days/week) |  |  |
| None | Reference | Reference |
| 1-2 times | 1.004 (0.935-1.077) | 0.997 (0.921-1.078) |
| 3-4 times | 0.984 (0.866-1.120) | 0.972 (0.843-1.120) |
| ≥ 5 times | 1.200 (1.039-1.385) | 1.257 (1.076-1.469) |
| Regular physical activity (days/week) |  |  |
| None | Reference | Reference |
| 1-4 days | 0.892 (0.839-0.949) | 0.887 (0.828-0.950) |
| ≥ 5 days | 0.973 (0.895-1.058) | 0.938 (0.856-1.028) |
| Comorbidities |  |  |
| Hypertension | 1.008 (0.948-1.073) | 1.017 (0.950-1.088) |
| Diabetes mellitus | 1.226 (1.123-1.338) | 1.203 (1.092-1.324) |
| Dyslipidemia | 1.148 (1.076-1.225) | 1.143 (1.065-1.226) |
| Stroke | 1.843 (1.338-2.538) | 1.990 (1.397-2.836) |
| Myocardial Infarction | 0.764 (0.342-1.704) | 0.567 (0.212-1.515) |
| COPD | 1.223 (1.154-1.297) | 1.232 (1.156-1.312) |
| Renal disease | 1.063 (0.930-1.215) | 1.083 (0.931-1.259) |
| Liver disease | 1.292 (1.210-1.380) | 1.302 (1.212-1.398) |
| Cancer | 1.051 (0.942-1.173) | 1.023 (0.902-1.161) |
| SDoH | 1.553 (1.350-1.786) | 1.515 (1.295-1.771) |
| IBD | 1.298 (1.109-1.519) | 1.235 (1.036-1.471) |
| Charlson comorbidity index |  |  |
| 0 | Reference | Reference |
| 1 | 1.027 (0.927-1.138) | 1.032 (0.922-1.156) |
| 2 or more | 0.883 (0.636-1.226) | 0.891 (0.635-1.252) |

PSM, propensity score matching; N, number; HR, hazard ratio; CI, confidence interval; COPD, chronic obstructive pulmonary disease; SDoH, social determinants of health; IBD, inflammatory bowel disease.

**Supplementary Table 11.** Results of Cox regression analysis for the association of surgical procedure/treatment for hemorrhoidal disease with incidence risk of Alzheimer’s disease.

| Variables | Before PSM N=24,489 | After 1:1 PSM N=18,330 |
| --- | --- | --- |
|  | Adjusted  HR (95%CI) | Adjusted  HR (95%CI) |
| Without treatment | Reference | Reference |
| With treatment | 0.911 (0.837-0.992) | 0.901 (0.823-0.987) |
| Age, years | 1.143 (1.137-1.149) | 1.144 (1.137-1.150) |
| Sex |  |  |
| Male | Reference | Reference |
| Female | 1.394 (1.259-1.543) | 1.405 (1.257-1.570) |
| Body mass index (kg/m^2^) | 0.988 (0.973-1.002) | 0.985 (0.969-1.001) |
| Household income |  |  |
| Low | Reference | Reference |
| Middle | 0.912 (0.820-1.014) | 0.903 (0.805-1.013) |
| High | 0.775 (0.695-0.865) | 0.788 (0.700-0.888) |
| Smoking status |  |  |
| Never | Reference | Reference |
| Former | 1.007 (0.856-1.185) | 1.029 (0.862-1.229) |
| Current | 1.072 (0.935-1.229) | 1.065 (0.916-1.239) |
| Alcohol consumption (days/week) |  |  |
| None | Reference | Reference |
| 1-2 times | 0.946 (0.842-1.064) | 0.954 (0.839-1.085) |
| 3-4 times | 1.029 (0.841-1.259) | 0.947 (0.756-1.187) |
| ≥ 5 times | 1.302 (1.065-1.591) | 1.382 (1.112-1.717) |
| Regular physical activity (days/week) |  |  |
| None | Reference | Reference |
| 1-4 days | 0.841 (0.760-0.931) | 0.825 (0.738-0.921) |
| ≥ 5 days | 0.956 (0.844-1.084) | 0.913 (0.796-1.047) |
| Comorbidities |  |  |
| Hypertension | 1.060 (0.967-1.162) | 1.075 (0.973-1.188) |
| Diabetes mellitus | 1.217 (1.070-1.385) | 1.185 (1.026-1.367) |
| Dyslipidemia | 1.084 (0.983-1.196) | 1.068 (0.960-1.189) |
| Stroke | 2.718 (1.890-3.910) | 2.902 (1.935-4.351) |
| Myocardial Infarction | 0.825 (0.265-2.568) | 0.584 (0.145-2.345) |
| COPD | 1.255 (1.149-1.371) | 1.300 (1.182-1.430) |
| Renal disease | 1.111 (0.918-1.344) | 1.193 (0.962-1.480) |
| Liver disease | 1.228 (1.111-1.356) | 1.275 (1.144-1.422) |
| Cancer | 1.020 (0.871-1.196) | 0.931 (0.768-1.128) |
| SDoH | 1.310 (1.064-1.612) | 1.300 (1.030-1.640) |
| IBD | 1.245 (0.985-1.573) | 1.161 (0.893-1.509) |
| Charlson comorbidity index |  |  |
| 0 | Reference | Reference |
| 1 | 0.918 (0.779-1.081) | 0.898 (0.750-1.076) |
| 2 or more | 1.029 (0.664-1.594) | 1.020 (0.649-1.603) |

PSM, propensity score matching; N, number; HR, hazard ratio; CI, confidence interval; COPD, chronic obstructive pulmonary disease; SDoH, social determinants of health; IBD, inflammatory bowel disease.

**Supplementary Table 12.** Results of Cox regression analysis for the association of surgical procedure/treatment for hemorrhoidal disease with incidence risk of vascular dementia.

| Variables | Before PSM N=24,489 | After 1:1 PSM N=18,330 |
| --- | --- | --- |
|  | Adjusted  HR (95%CI) | Adjusted  HR (95%CI) |
| Without treatment | Reference | Reference |
| With treatment | 0.865 (0.758-0.988) | 0.848 (0.736-0.976) |
| Age, years | 1.107 (1.099-1.116) | 1.111 (1.102-1.121) |
| Sex |  |  |
| Male | Reference | Reference |
| Female | 1.118 (0.956-1.307) | 1.145 (0.964-1.361) |
| Body mass index (kg/m^2^) | 1.031 (1.009-1.055) | 1.023 (0.998-1.048) |
| Household income |  |  |
| Low | Reference | Reference |
| Middle | 0.970 (0.823-1.144) | 0.995 (0.832-1.190) |
| High | 0.804 (0.677-0.955) | 0.799 (0.662-0.965) |
| Smoking status |  |  |
| Never | Reference | Reference |
| Former | 1.089 (0.862-1.376) | 1.084 (0.839-1.402) |
| Current | 1.086 (0.886-1.331) | 1.087 (0.868-1.360) |
| Alcohol consumption (days/week) |  |  |
| None | Reference | Reference |
| 1-2 times | 1.028 (0.865-1.221) | 1.023 (0.846-1.238) |
| 3-4 times | 0.756 (0.539-1.059) | 0.783 (0.544-1.125) |
| ≥ 5 times | 1.442 (1.077-1.930) | 1.415 (1.025-1.955) |
| Regular physical activity (days/week) |  |  |
| None | Reference | Reference |
| 1-4 days | 0.843 (0.723-0.983) | 0.887 (0.750-1.050) |
| ≥ 5 days | 0.954 (0.784-1.160) | 0.975 (0.788-1.206) |
| Comorbidities |  |  |
| Hypertension | 1.242 (1.079-1.429) | 1.293 (1.110-1.505) |
| Diabetes mellitus | 1.469 (1.222-1.767) | 1.391 (1.131-1.710) |
| Dyslipidemia | 1.013 (0.869-1.180) | 1.035 (0.878-1.222) |
| Stroke | 3.133 (1.924-5.102) | 3.716 (2.182-6.329) |
| Myocardial Infarction | 0.262 (0.016-4.208) | 0.297 (0.019-4.768) |
| COPD | 1.093 (0.952-1.256) | 1.109 (0.955-1.288) |
| Renal disease | 1.212 (0.919-1.599) | 1.171 (0.851-1.612) |
| Liver disease | 1.292 (1.109-1.506) | 1.341 (1.136-1.583) |
| Cancer | 0.972 (0.756-1.249) | 0.850 (0.623-1.159) |
| SDoH | 1.260 (0.908-1.748) | 1.226 (0.846-1.775) |
| IBD | 1.240 (0.868-1.770) | 1.391 (0.952-2.033) |
| Charlson comorbidity index |  |  |
| 0 | Reference | Reference |
| 1 | 0.868 (0.667-1.130) | 0.789 (0.585-1.065) |
| 2 or more | 1.502 (0.839-2.688) | 1.684 (0.934-3.038) |

PSM, propensity score matching; N, number; HR, hazard ratio; CI, confidence interval; COPD, chronic obstructive pulmonary disease; SDoH, social determinants of health; IBD, inflammatory bowel disease.

**Supplementary Table 13.** Wald Chi-square test results from the Cox model by treatment status (before propensity score matching)

|  |  |  | All-cause dementia | | Alzheimer’s disease | | Vascular dementia | |
| --- | --- | --- | --- | --- | --- | --- | --- | --- |
| Variables | Test Type | DF | Wald χ² | P-value | Wald χ² | P-value | Wald χ² | P-value |
| Unadjusted Model |  |  |  |  |  |  |  |  |
| Overall Model | Global | 1 | 236.124 | <0.001 | 175.523 | <0.001 | 69.802 | <0.001 |
| Adjusted Model |  |  |  |  |  |  |  |  |
| Overall Model | Global | 26 | 5485.350 | <0.001 | 3835.740 | <0.001 | 1198.580 | <0.001 |
| With treatment | Type3 | 1 | 12.701 | <0.001 | 4.432 | 0.035 | 4.656 | 0.034 |
| Age, years | Type3 | 1 | 2629.411 | <0.001 | 2709.956 | <0.001 | 710.777 | <0.001 |
| Sex | Type3 | 1 | 117.061 | <0.001 | 41.051 | <0.001 | 1.943 | 0.163 |
| Body mass index (kg/m^2^) | Type3 | 1 | 1.278 | 0.258 | 2.757 | 0.096 | 7.315 | 0.006 |
| Household income | Type3 | 2 | 27.620 | <0.001 | 21.549 | <0.001 | 7.828 | 0.020 |
| Smoking status | Type3 | 2 | 1.448 | 0.484 | 1.019 | 0.601 | 0.913 | 0.633 |
| Alcohol consumption (days/week) | Type3 | 3 | 6.623 | 0.036 | 8.947 | 0.011 | 10.151 | 0.006 |
| Regular physical activity (days/week) | Type3 | 2 | 13.168 | 0.001 | 11.232 | 0.003 | 4.734 | 0.093 |
| Hypertension | Type3 | 1 | 0.071 | 0.789 | 1.532 | 0.215 | 9.165 | 0.002 |
| Diabetes mellitus | Type3 | 1 | 20.713 | <0.001 | 8.918 | 0.002 | 16.735 | <0.001 |
| Dyslipidemia | Type3 | 1 | 17.542 | <0.001 | 2.615 | 0.106 | 0.027 | 0.870 |
| Stroke | Type3 | 1 | 16.374 | <0.001 | 29.082 | <0.001 | 21.080 | <0.001 |
| Myocardial Infarction | Type3 | 1 | 0.431 | 0.511 | 0.110 | 0.739 | 0.882 | 0.347 |
| COPD | Type3 | 1 | 45.891 | <0.001 | 25.400 | <0.001 | 1.737 | 0.187 |
| Renal disease | Type3 | 1 | 0.799 | 0.371 | 1.175 | 0.278 | 1.012 | 0.314 |
| Liver disease | Type3 | 1 | 59.008 | <0.001 | 16.222 | <0.001 | 10.815 | 0.001 |
| Cancer | Type3 | 1 | 0.793 | 0.373 | 0.060 | 0.806 | 0.049 | 0.824 |
| SDoH | Type3 | 1 | 37.931 | <0.001 | 6.475 | 0.010 | 1.912 | 0.166 |
| IBD | Type3 | 1 | 10.553 | 0.001 | 3.362 | 0.066 | 1.397 | 0.237 |
| Charlson comorbidity index | Type3 | 2 | 0.829 | 0.660 | 1.084 | 0.581 | 3.082 | 0.214 |

DF, degrees of freedom; COPD, chronic obstructive pulmonary disease; SDoH, social determinants of health; IBD, inflammatory bowel disease.

S13 Table presents the Wald Chi-square test results from the Cox proportional hazards model including hemorrhoid treatment status as a covariate, based on the cohort before propensity score matching (N = 24,489). The global test confirms overall model significance, and Type 3 tests assess the effect of each covariate including treatment.

**Supplementary Table 14.** Wald Chi-square test results from the Cox model by treatment status (after propensity score matching)

|  |  |  | All-cause dementia | | Alzheimer’s disease | | Vascular dementia | |
| --- | --- | --- | --- | --- | --- | --- | --- | --- |
| Variables | Test Type | DF | Wald χ² | P-value | Wald χ² | P-value | Wald χ² | P-value |
| Unadjusted Model |  |  |  |  |  |  |  |  |
| Overall Model | Global | 1 | 4.445 | 0.035 | 4.668 | 0.030 | 5.370 | 0.020 |
| Adjusted Model |  |  |  |  |  |  |  |  |
| Overall Model | Global | 26 | 3900.261 | <0.001 | 2881.332 | <0.001 | 902.448 | <0.001 |
| With treatment | Type3 | 1 | 6.492 | 0.010 | 4.937 | 0.026 | 5.068 | 0.024 |
| Age, years | Type3 | 1 | 2774.943 | <0.001 | 2181.598 | <0.001 | 601.062 | <0.001 |
| Sex | Type3 | 1 | 90.450 | <0.001 | 35.826 | <0.001 | 2.357 | 0.124 |
| Body mass index (kg/m^2^) | Type3 | 1 | 0.442 | 0.505 | 3.447 | 0.063 | 3.313 | 0.068 |
| Household income | Type3 | 2 | 22.131 | <0.001 | 15.517 | <0.001 | 7.780 | 0.020 |
| Smoking status | Type3 | 2 | 1.070 | 0.585 | 0.690 | 0.708 | 1.086 | 0.581 |
| Alcohol consumption (days/week) | Type3 | 3 | 9.285 | 0.009 | 6.194 | 0.045 | 7.265 | 0.026 |
| Regular physical activity (days/week) | Type3 | 2 | 12.019 | 0.002 | 11.869 | 0.002 | 1.973 | 0.372 |
| Hypertension | Type3 | 1 | 0.233 | 0.629 | 2.040 | 0.153 | 10.896 | 0.001 |
| Diabetes mellitus | Type3 | 1 | 14.142 | <0.001 | 5.366 | 0.020 | 9.811 | 0.001 |
| Dyslipidemia | Type3 | 1 | 13.867 | <0.001 | 1.457 | 0.227 | 0.170 | 0.680 |
| Stroke | Type3 | 1 | 14.508 | <0.001 | 26.556 | <0.001 | 23.336 | <0.001 |
| Myocardial Infarction | Type3 | 1 | 1.279 | 0.258 | 0.576 | 0.448 | 0.749 | 0.386 |
| COPD | Type3 | 1 | 41.245 | <0.001 | 29.251 | <0.001 | 1.840 | 0.175 |
| Renal disease | Type3 | 1 | 1.065 | 0.301 | 2.572 | 0.108 | 0.944 | 0.331 |
| Liver disease | Type3 | 1 | 52.503 | <0.001 | 19.258 | <0.001 | 12.091 | <0.001 |
| Cancer | Type3 | 1 | 0.125 | 0.723 | 0.530 | 0.466 | 1.051 | 0.305 |
| SDoH | Type3 | 1 | 26.927 | <0.001 | 4.879 | 0.027 | 1.159 | 0.281 |
| IBD | Type3 | 1 | 5.544 | 0.018 | 1.243 | 0.264 | 2.909 | 0.088 |
| Charlson comorbidity index | Type3 | 2 | 0.768 | 0.681 | 1.373 | 0.503 | 5.563 | 0.061 |

DF, degrees of freedom; COPD, chronic obstructive pulmonary disease; SDoH, social determinants of health; IBD, inflammatory bowel disease.

S14 Table presents the Wald Chi-square test results from the Cox proportional hazards model including hemorrhoid treatment status as a covariate, based on the cohort after propensity score matching (N = 18,330). The global test confirms overall model significance, and Type 3 tests assess the effect of each covariate including treatment.

**Supplementary Table 15**. Results of the mediation analysis on the association between hemorrhoidal disease and the incidence risk of dementia, considering SDoH as a mediator.

| Variable | Before PSM | After 1:5 PSM |
| --- | --- | --- |
|  | N = 358,139 | N = 138,792 |
|  | Adjusted | Adjusted |
|  | HR (95%CI) | HR (95%CI) |
| All-cause dementia |  |  |
| NDE (c) | 1.228 (1.191–1.267) | 1.236 (1.202–1.271) |
| NIE (a×b) | 1.005 (1.003–1.006) | 1.006 (1.004–1.008) |
| Alzheimer’s disease |  |  |
| NDE (c) | 1.076 (1.031–1.123) | 1.099 (1.047–1.155) |
| NIE (a×b) | 1.005 (1.003–1.006) | 1.006 (1.003–1.008) |
| Vascular dementia |  |  |
| NDE (c) | 1.130 (1.068–1.196) | 1.160 (1.089–1.237) |
| NIE (a×b) | 1.105 (1.103–1.107) | 1.107 (1.104–1.110) |

SDoH, social determinants of health; PSM, propensity score matching; N, number; HR, hazard ratio; CI, confidence interval; NDE, natural direct effect; NIE, natural indirect effect.

(a) and (b×c) represent the relationships illustrated in S1 Figure.

**Supplementary Table 16**. Results of the mediation analysis for the association of surgical procedure/treatment for hemorrhoidal disease with incidence risk of dementia, considering SDoH as a mediator.

| Variable | Before PSM | After 1:1 PSM |
| --- | --- | --- |
|  | N = 24,489 | N = 18,330 |
|  | Adjusted | Adjusted |
|  | HR (95%CI) | HR (95%CI) |
| All-cause dementia |  |  |
| NDE (c) | 0.907 (0.853–0.965) | 0.928 (0.875–0.985) |
| NIE (a×b) | 0.994 (0.991–0.998) | 0.996 (0.993–0.999) |
| Alzheimer’s disease |  |  |
| NDE (c) | 0.913 (0.837–0.995) | 0.904 (0.824–0.993) |
| NIE (a×b) | 0.996 (0.992–0.999) | 0.998 (0.995–0.999) |
| Vascular dementia |  |  |
| NDE (c) | 0.869 (0.760–0.994) | 0.850 (0.738–0.981) |
| NIE (a×b) | 0.967 (0.937–0.997) | 0.998 (0.996–0.999) |

SDoH, social determinants of health; PSM, propensity score matching; N, number; HR, hazard ratio; CI, confidence interval; NDE, natural direct effect; NIE, natural indirect effect.

(c) and (a×b) represent the relationships illustrated in S1 Figure.

**Supplementary Table 17.** Results of GVIF for covariates.

| Variables | Hemorrhoidal disease and all-cause dementia | | | Surgical procedure/treatment for hemorrhoidal disease and all-cause dementia | | |
| --- | --- | --- | --- | --- | --- | --- |
|  | GVIF | DF | GVIF^1/2df^ | GVIF | DF | GVIF^1/2df^ |
| Age, years | 1.145 | 1 | 1.070 | 1.158 | 1 | 1.076 |
| Sex | 1.641 | 1 | 1.281 | 1.534 | 1 | 1.238 |
| Body mass index (kg/m^2^) | 1.071 | 1 | 1.035 | 1.070 | 1 | 1.034 |
| Household income | 1.360 | 2 | 1.080 | 1.473 | 1 | 1.102 |
| Smoking status | 1.184 | 2 | 1.043 | 1.185 | 2 | 1.043 |
| Alcohol consumption (days/week) | 1.279 | 3 | 1.042 | 1.292 | 2 | 1.044 |
| Regular physical activity (days/week) | 1.135 | 2 | 1.032 | 1.095 | 3 | 1.046 |
| Hypertension | 1.090 | 1 | 1.044 | 1.095 | 2 | 1.046 |
| Diabetes mellitus | 1.056 | 1 | 1.028 | 1.048 | 1 | 1.024 |
| Dyslipidemia | 1.221 | 1 | 1.105 | 1.237 | 1 | 1.112 |
| Stroke | 1.006 | 1 | 1.003 | 1.006 | 1 | 1.003 |
| Myocardial Infarction | 1.005 | 1 | 1.003 | 1.006 | 1 | 1.003 |
| COPD | 1.047 | 1 | 1.023 | 1.050 | 1 | 1.025 |
| Renal disease | 1.057 | 1 | 1.028 | 1.056 | 1 | 1.028 |
| Liver disease | 1.164 | 1 | 1.079 | 1.167 | 1 | 1.080 |
| Cancer | 1.059 | 1 | 1.029 | 1.055 | 1 | 1.027 |
| SDoH | 1.059 | 1 | 1.029 | 1.078 | 1 | 1.038 |
| IBD | 1.005 | 1 | 1.003 | 1.006 | 1 | 1.003 |
| Charlson comorbidity index | 1.016 | 1 | 1.008 | 1.017 | 1 | 1.008 |

GVIF, generalized variance inflation factor; DF, the number of degrees of freedom; COPD, chronic obstructive pulmonary disease; SDoH, social determinants of health; IBD, inflammatory bowel disease.

This analysis is a multicollinearity analysis of covariates used in the Cox regression models for the associations between hemorrhoidal disease and all-cause dementia, as well as surgical procedures/treatments for hemorrhoidal disease and all-cause dementia.
